# Do drinking water plants retain microplastics? An exploratory study using Raman micro-spectroscopy^☆^

**DOI:** 10.1016/j.heliyon.2023.e17113

**Published:** 2023-06-09

**Authors:** Luca Maurizi, Lucian Iordachescu, Inga V. Kirstein, Asbjørn H. Nielsen, Jes Vollertsen

**Affiliations:** a Department of the Built Environment, Aalborg University, Thomas Manns Vej 23, 9220, Aalborg, Denmark; b Alfred-Wegener-Institute Helmholtz Centre for Polar and Marine Research, Biologische Anstalt Helgoland, Helgoland, Germany

**Keywords:** Microplastics, Nanoplastics, Raman micro-spectroscopy, Drinking water, Water quality, Plastic pollution

## Abstract

The retainment of microplastics (MPs) down to 1 μm by a Danish drinking water plant fed with groundwater was quantified using Raman micro-spectroscopy (μRaman). The inlet and outlet were sampled in parallel triplicates over five consecutive days of normal activity. For each triplicate, approximately 1 m^3^ of drinking water was filtered with a custom-made device employing 1 μm steel filters. The MP abundance was expressed as MP counts per liter (N/L) and MP mass per liter (pg/L), the latter being estimated from the morphological parameters provided by the μRaman analysis. Hence the treated water held on average 1.4 MP counts/L, corresponding to 4 pg/L. The raw water entering the sand filters held a higher MP abundance, and the overall efficiency of the treatment was 43.2% in terms of MP counts and 75.1% in terms of MP mass. The reason for the difference between count-based and mass-based efficiencies was that 1–5 μm MP were retained to a significantly lower degree than larger ones. Above 10 μm, 79.6% of all MPs were retained by the filters, while the efficiency was only 41.1% below 5 μm. The MP retainment was highly variable between measurements, showing an overall decreasing tendency over the investigated period. Therefore, the plastic elements of the plant (valves, sealing components, etc.) likely released small-sized MPs due to the mechanical stress experienced during the treatment. The sub-micron fraction (0.45–1 μm) of the samples was also qualitatively explored, showing that nanoplastics (NPs) were present and that at least part hereof could be detected by μRaman.

## Highlights

• Retainment of MPs >1 μm by a drinking waterwork investigated with μRaman.
• Sand filtration overall effective, although deteriorating for MPs <5 μm.
• Aged plastic parts within the facility potentially releasing small-sized MPs.
• Human intake from the studied drinking water: 1533 MP s/(year·capita).
• Qualitative exploration of the NP fraction 0.45–1 μm.

## Graphical abstract

**Figure:**
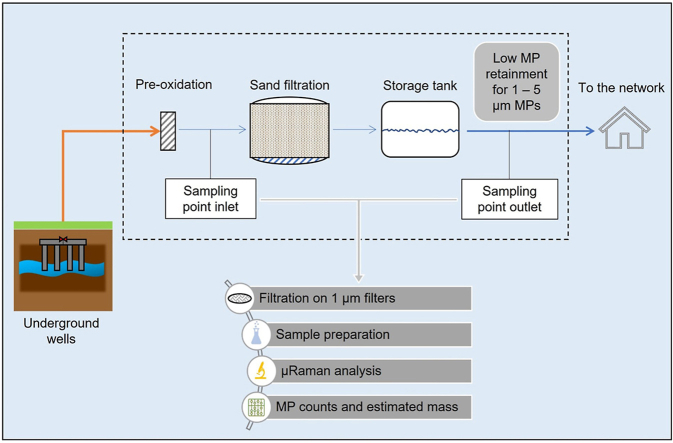

## 1 Introduction

Humans are exposed to microplastics (MPs) and nanoplastics (NPs) with consequences still unclear [1]. Contact with MPs and NPs may happen *via* pathways such as air [2], water [3], and food [4]. Consequently, the human organism is continuously subjected to plastic fragments below the millimetric size [5], which, for example, has been confirmed by analyzing human stool and placentas ([6,7]).

By implicit yet unofficial convention, plastic particles smaller than 1 μm have been termed *nanoplastics* [8], while *microplastics* span between 1 μm and 5 mm in size. This categorization was also followed in the present paper. The MP category may be further divided into “large microplastics” (above 1 mm) and “small microplastics” (below 1 mm) [9], as well as “primary microplastics” (purposely added to cosmetics and personal-care products) and “secondary microplastics” (produced by plastic objects breaking down in the environment) [10].

A report published by the World Health Organization (WHO) in 2019 claimed that there is no evidence that MPs in drinking water could be harmful to humans [11]. However, the same report stressed the lack of a comprehensive overview of MP and NP occurrence in drinking water plants and urban water networks. At the same time, a growing number of studies have pointed out that MPs and NPs can have a wide range of biological effects [12] on plants [13] and animals [14] in their habitats. Although most of the studies published so far address marine species as models for MP and NP toxicity, a cautionary approach would suggest also studying the exposure of humans [15]. In general, MPs and NPs are believed to increase their toxicity as they get smaller ([16,17]), probably due to translocation across biological membranes such as intestines and accumulation in tissue [18]. Different exposure routes for humans are possible, drinking water being one of them [19]. As a response to this, the European Union has recently put microplastics on their watch list for the quality of water intended for human consumption [20].

Drinking water can be distinguished according to its source, namely groundwater and surface water [21]. The MP analysis may be conducted either on the raw water ([[22], [23], [24]]), after the water treatment, or at the inlet and outlet of a waterworks ([[25], [26], [27]]). The latter also enables estimating the plant's removal efficiency for MPs within a certain size range. Generally implementing more treatment steps improves the MP removal [28], hence drinking water plants equipped only with coagulation-flocculation-sedimentation (CFS) and sand filtration tend to show rather low MP retainment efficiency (∼40%) [28]. In contrast, more advanced waterworks with CFS-sand filtration-ozonation-granular active carbon (GAC) filtration can reach ∼80% of MP removal [28]. The performance of each treatment step seems influenced by the size, shape, polymer type, surface electric charge of the MPs in the raw water, and, additionally, by the chemicals employed in the treatment. For CFS, the choice of polyaluminium chloride (PACl) as coagulant has led to higher MP removal than ferric chloride (FeCl_3_·6H_2_O) in laboratory-scale tests [29], probably thanks to stronger electrostatic interactions between the MPs and coagulant flocs. While fibres are reportedly better retained than fragments in CFS [30], the role of MP size is still under debate, as the literature provides contrasting findings. After CFS, media filtration is normally performed, however, the MP retainment reached in this step depends on the media employed. [31] saw that 5–10 μm MP can elude sand filters, therefore additional treatments should be implemented after sand filtration. GAC seems a valid complement to sand filtration and has been shown to remove up to 61% of the residual MPs in the water, despite losing efficiency for fibrous shapes [32]. Transport, attachment, and detachment are the main mechanisms involved in media filtration of particles [33], and, according to granular media filtration theories, particles close to 1 μm exhibit the minimum net transport efficiency [34]. However, dedicated studies on the effect of different porous media filtration conditions on MP removal lack, hence only indirect insights can be drawn from past works. Besides CFS and porous media filtration, the impact of ozonation on MP removal was also investigated [32]. After the ozonation step, the MP concentration slightly increased, possibly because of the breakdown of larger MPs into smaller particles.

The most widely employed spectroscopic methods for environmental MP analysis are Fourier-Transform Infrared micro-spectroscopy (μFTIR) and Raman micro-spectroscopy (μRaman). Schymanski et al. [35] underlined the urgent need for improving harmonization and reproducibility among the protocols based on these techniques. A plea similar to that of [35] was expressed by Kirstein et al. [36], regarding MP toxicology assessment and MP quantification in drinking water.

The aim of this work is to assess whether and to what extent a drinking water plant fed by groundwater can retain MPs down to 1 μm, preventing them to reach the distribution network. Despite being a more protected environment than surface waters, groundwater sources can indeed also be affected by MP pollution [37]. The study assesses the MP retainment by comparing two MP parameters: counts and mass, the latter being estimated from the morphological data provided by the μRaman analysis. Currently, mass estimation of MPs and NPs is mostly performed with Pyr-GC/MS [38], which provides quantitatively accurate results. However, it does not provide information about particle size distribution and morphology. In addition, its quantification limit is roughly in the order of 1 μg per sample and polymer type, of course depending on the polymer to be quantified and the instrumentation used. The mass of small (<10 μm) MPs and NPs in a drinking water sample may however be orders of magnitude below such a detection limit. There is therefore a need for an analytical method capable of providing more information on small MPs and NPs, their chemical identity, size, abundance as well as mass in a reasonable time and within one analytical run.

## 2 Materials and methods

### 2.1 Drinking water sampling

The investigated plant produces approximately 500,000 m^3^/year of drinking water and it is located in North Jutland. The raw water is extracted from a limestone aquifer (4 wells with screens 72–92 m below ground) using submersible pumps and treated through aeration and rapid sand filtration (see also Supplementary Information, Fig. S1). The closed environment of the aquifer is not affected by seasonal variations, having an age of many years. From the wells, the water is conveyed approximately 800 m in high-density poly-ethylene (HDPE) pipes to the waterworks, whereafter it is transported in steel pipes between the individual process steps of the facility. Before being sent to the water distribution network, the treated water is stored in two stainless steel tanks of 110 m^3^ each (daytime retention time 2–3 h).

Two custom made devices were employed on Series 1 of the facility, which operates symmetrically. Each device had 4 parallel flow lines holding 1 μm sintered steel filters (Mesh Masters, The Netherlands) of 46 mm diameter. One device was placed at the inlet before the sand filters and one at the outlet after the storage tanks. The sampling devices were connected at the sampling points by flexible steel pipes and the plant was sampled daily between the 27th of September and the 1st of October 2021 (9 a.m.–2 p.m.). Approx. 1 m^3^ of drinking water was filtered on each of the parallel operated filters along the first three flow lines of the two devices [39]. Inlet and outlet were sampled in parallel, resulting in a total of 5 inlet and 5 outlet samples collected in triplicate (n = 30). A flowmeter mounted at the outlet of each flow line measured the filtered volume for each filter. At the beginning of each sampling, the flow per line was adjusted to approximately 3 L/min.

Field blanks were collected in parallel with the drinking water samples by equipping the fourth flow line of each device with two filter holders coupled in series. The first held a muffled 0.7 μm glass fibre membrane (Th. Geyer GmbH, Germany), and the second a 1 μm sintered steel filter. The glass-fibre membrane served as a pre-filter to sample the drinking water matrix while excluding the MPs above 0.7 μm [39]. For each field blank, the filtered volume was recorded by a flowmeter at the outlet of the flowline, and the average flow was adjusted to 3 L/min. Contamination occurs during the preparation stage of the filters and during the analysis, but not during the sampling itself, where the filter is isolated from the surroundings. Consequently, the degree of contamination of a field blank is independent of the sampled volume, and it was chosen to filter approx. 20 L for each field blank (n = 10). After the sampling, the steel filters of the drinking water samples and the field blanks were stored in Petri dishes prior to the sample preparation. Further details on the sampling device are given in Supplementary Information.

### 2.2 Preparation of recovery samples

Standard plastic fragments (poly-ethylene 45–100 μm, poly-styrene 10–40 μm, and poly-ethylene terephthalate 10–40 μm, GoodFellow Corp., USA) were chosen to provide a recovery assessment comparable with data commonly reported in the literature. The particles were added to three clean beakers with 5 mL Milli-Q water (Type 1 ultrapure water, 18.2 MΩ cm) (Purelab Ultra Elga, UK) and counted with a FlowCam (Yokogawa Fluid Imaging Technologies, Inc., USA). The three suspensions with a known amount of each MP standard were filtered through a 1 μm sintered steel filter each with a vacuum filtration system. The so-enriched filters were placed in one of the sampling devices used at the waterworks. Similar to what was done with the field blanks, a filter holder with a muffled 0.7 μm glass fibre membrane was placed before each enriched steel filter and 1 m^3^ of tap water was filtered through each flow line. The purpose of this procedure was to reproduce the sampling conditions without adding further MP above 0.7 μm to the recovery samples. The flow was adjusted to approximately 3 L/min per flow line. Finally, the enriched steel filters were stored in Petri dishes prior to sample preparation.

### 2.3 Contamination prevention

The steel filters were sonicated in Milli-Q water (18.2 MΩ cm), dried at 50 °C in an oven, and stored in muffled Petri dishes in a laminar flow bench before use. All chemicals were filtered through muffled 0.7 μm glass fibre membranes. All glassware was muffled at 500 °C for 4 h, and metallic labware was thoroughly rinsed with Milli-Q water. All sample preparation took place in a laminar flow bench, which was regularly cleaned with 50% EtOH. Pure cotton lab coats were worn during the entire sample preparation.

### 2.4 Sample preparation and analysis

All samples (drinking water samples, field blanks, and recovery samples) were processed following a protocol similar to that employed by Ref. [39]. In brief: each filter was incubated for 24 h in 5% SDS at 50 °C, upon which the particles were transferred to the SDS solution by ultrasonication. The particle-enriched mixture was then filtered through the same filter on which the sample originally was collected. Hereafter the particles were sonicated from the filter into 50% EtOH, and the filtration procedure repeated with the ethanolic particle-enriched solution. Finally, the filter was immersed in 99.5% EtOH, ultrasonicated, and scratched with a metallic spatula to collect all particles. The ethanolic mixture was poured into a 10 mL vial and evaporated under nitrogen flow until exhaustion.

Sample reconstitution, deposition, and analysis took place at Renishaw plc UK (Wotton-under-Edge, UK). Samples were reconstituted with 1.0 mL of >99.5% EtOH in a flow bench. Upon reconstitution, neither sedimentation nor flotation of particles could be observed, and the mixtures were quite clear. Immediately before the deposition, the ethanolic mixture was homogenized with a vortex mixer (VWR, Denmark) for 30 s. Then, 25.0 μL from each vial were deposed on a 10 × 10 mm single-crystal silicon (Si) substrate (SmartMembranes GmbH, Germany), using a manual micropipette mounting a 25.0 μL glass tip (Brand GmbH, Germany). The deposition was restricted to a circular area of 2 mm diameter by a custom-made device, obtaining an active area of approximately 3.14·10^6^ μm^2^ for each sample. Finally, the samples were dried at 55 °C overnight on a heating plate (W10 VWR, Denmark). Depositing the 2.5% of each sample allowed for obtaining active areas with no particles covering each other and with an average number of MPs analyzable without employing any sort of sub-mapping, i.e., the full area was analyzed by the Raman microscope in one session.

**Fig. 1:**
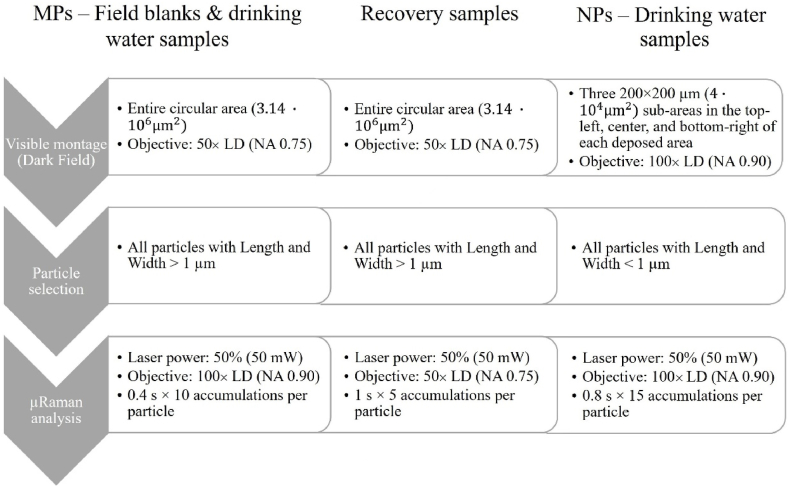
Scheme of the analysis protocol followed for the field blanks, drinking water samples, and recovery samples.

The employed Raman microscope was a Renishaw InVia equipped with a 1024 × 256 pixel CCD detector cooled at −70 °C and a 532 nm Nd: YAG solid-state laser source (Renishaw plc UK, UK). The software module “Particle Analysis” (Renishaw plc UK, UK) was used to perform the analysis of the visible image, the μRaman analysis, and the spectral processing and recognition. For the μRaman analysis, a grating density of 1800 ll/mm and two LD (long distance) objectives (Leica, Germany) 50 × (NA 0.75) and 100 × (NA 0.90) were chosen, and the spectral range was set to 300–2000 cm^−1^. The system was previously calibrated by zero-order correction on the first-order Raman emission of single-crystal Silicon at 519.5 ± 0.5 cm^−1^. Fig. 1 shows the analysis process followed for the samples. Briefly: after the acquisition of the visible image (montage), the software calculated the morphological parameters of the particles in the montage and saved their XY coordinates. The particles were virtually selected according to their size, and the laser was automatically driven onto each particle to obtain its Raman spectrum.

**Fig. 2:**
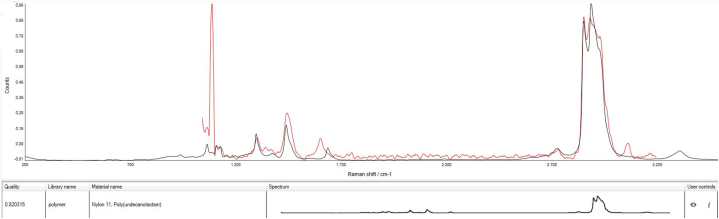
Raman spectrum of Nylon 11 MP (category PA), also showing a peak belonging to bicarbonate (∼1000 cm-1) (HQ 0.82).

**Fig. 3:**
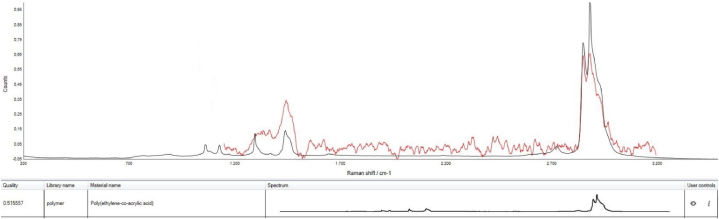
Raman spectrum of PE nanoparticle (HQ 0.51).

The environmental particles in the analyzed active areas looked transparent, and no fluorescence was encountered during the spectral acquisition. The μRaman spectra were baseline-corrected and compared with the Renishaw Polymer Library, and only spectra with hit quality (HQ, quality of the fit result between the experimental spectrum and the model composed by a linear combination of reference spectra from the library) of at least 80% were considered for the MP quantification [35]. Accordingly, the particles not fulfilling this criterium were considered non-plastic. For the recovery samples, the HQ threshold could be reached at a lower magnification objective during the spectral acquisition, given the larger mean size of the fragments employed for the recovery test in comparison with the mean size of the MPs found in the field blanks and drinking water samples. For the qualitative analysis of the NPs, a HQ above 50% was considered sufficient. Fig. 2, Fig. 3 are examples of experimental spectra from an MP and NP, respectively.

Further details, pictures, and examples of spectra are given in Supplementary Information.

### 2.5 Post-processing of the μRaman data

The MP mass from the drinking water samples and field blanks was estimated from the morphological parameters of the particles in the active areas. The analysis of the visible montage yielded the area of the 2D projection and the length (L) of each particle. Then, the width of the equivalent ellipse (W_eq_) was calculated from the area and the length, and it was assumed that the third diameter (perpendicular to the remaining two) was 60% of the W_eq_ [40]. Hence the volume (V) of each particle was calculated according to equation (1):

(1) $$V=\left(4/3\right)\cdot \pi \cdot \left(L/2\right)\cdot \left(\;W_{eq}/2\right)\cdot \left(0.6\cdot W_{eq}/2\right)$$

Next, each plastic particle was assigned a pure polymer density according to its spectral identification from the μRaman analysis, which allowed for the particle mass in picograms (pg, 10^−12^ g) to be estimated.

The plant's removal efficiency R was calculated by comparing the cumulated MP counts or estimated mass of the inlet with that of the outlet (equation (2)):

(2) $$R=-\left(\left(\left[{MPs}_{outlet}\right]–\;\left[{MPs}_{inlet}\right]\right)/\left[{MPs}_{inlet}\right]\right)\cdot 100\%$$

Where [MPsinlet] and [MPsoutlet] respectively indicate the cumulated MP abundance in either counts (N/L) or mass (pg/L) at the plant's inlet and outlet. The cumulated MP abundance was calculated by adding the MP abundance of each investigated day at the plant's inlet or outlet.

LOD (Limit of Detection) and LOQ (Limit of Quantification) were calculated from the field blanks. They were calculated for total MP abundance and each identified polymer in the blanks, both for the MP counts and mass estimation. Equations (3), (4) were applied [35]:

(3) $$LOD={mean}_{blanks}+3\cdot s_{mean}$$

(4) $$LOQ={mean}_{blanks}+10\cdot s_{mean}$$

Where meanblanks is the mean count or mass of each polymer found in the 10 field blanks and smean the corresponding standard deviation. The mean blank value of each polymer was subtracted from the corresponding value determined in the sample, which was considered only if above the LOQ after the correction.

The recovery rate Q for each positive control was calculated as follows (equation (5)):

(5) $$Q=\left(N_{recovered}/N_{initial}\right)\;100\%$$

Where Nrecovered is the count of standard fragments recovered and normalized to the fraction of volume deposited, and Ninitial is the count of standard fragments initially added.

Uncertainty was calculated by selecting a coverage factor *k* = 2 (*P* = *95%*) and considering only the A-type (statistical) contribution. Statistical tests were performed with Rstudio v. 2022.02.1. ANOVA test was conducted for significance assessment (α = 0.05) and Tukey-test for pair-wise factor comparison. Normality was assessed with the Shapiro-Wilk test. The samples were labeled as Dxy, where D means “Day”, x is the sampling day (1–5), and y is either i for inlet or o for outlet.

## 3 Results and discussion

### 3.1 Field blanks, LOD, LOQ, and recovery

Although great care was exercised to avoid contamination from the environment, MPs were present in the blanks. The polymers identified in the blanks were common and widely used in laboratory items [25]. To relate the LOD and LOQ to a sampled volume, the LOD and LOQ per sample were divided by the volume filtered (approx. 1 m^3^). Hence LOD = 0.255 N/L and LOQ = 2.559 N/L for the abundance measured as counts, and LOD = 0.645 pg/L and LOQ = 1.957 pg/L for the abundance measured as mass.

The mean recovery rate for the MP counts was 91.7 ± 12.3% for the length range 10–120 μm, which is comparable with the value obtained by Weber et al. [41] (53 ± 14% for the 22–27 μm range and 89 ± 28% for the 45–53 μm range). Further details on LOD, LOQ, and MP recovery are given in section 4 of the Supplementary Information.

### 3.2 Removal at the plant

**Fig. 4:**
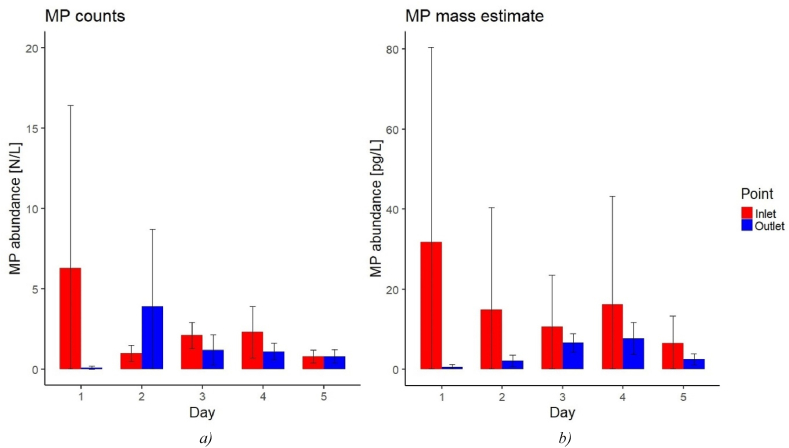
Overall MP abundance in a) counts per liter and b) estimated mass per liter at the plant's inlet and outlet for each investigated day. The error bars indicate the standard deviation for each day.

Fig. 4 shows the overall MP abundance at the inlet and outlet as counts per liter and pg per liter. The average inlet abundance was 2.5 N/L, corresponding to 16.0 pg/L, while the average outlet abundance was 1.4 N/L (4.0 pg/L). In terms of MP counts, the outlet on D1 (0.1 ± 0.1 N/L) was substantially below that of the inlet (6.3 ± 11.7 N/L), while it on D2 was approx. three times above that of the inlet (1.0 ± 0.6 N/L *versus* 3.9 ± 5.6 N/L). In terms of mass, MPs were retained on D2 (inlet 14.9 ± 29.5 pg/L, outlet 2.2 ± 1.7 pg/L), albeit to a lower degree than on D1 (inlet 31.8 ± 56.3 pg/L, outlet 0.6 ± 0.6 pg/L). Hence the counts showed a production of total MP over the treatment works, while the mass showed a retainment. Similarly, on D5 there was no net retainment in terms of counts (inlet 0.8 ± 0.4 N/L, outlet 0.8 ± 0.5 N/L), but there was a net retainment in terms of mass (inlet 6.5 ± 7.9 N/L, outlet 2.6 ± 1.5 N/L). Therefore, on those two days, the MPs in the outlet tended to hold less mass altogether than in the inlet, meaning that they were generally small-sized. In particular, the pre-aeration step before the sand filtration may have led to a first breakdown of the MPs in the raw groundwater, which may have been followed by a secondary degradation in the sand filters due to the friction between MPs, non-plastic particles, and the granular medium. In addition, the generally low retainment efficiency for MPs <10 μm characterizing sand filters, may have further enhanced the enrichment in small-sized MPs of the treated water. This hypothesis is also backed by the general overview of the effect of the different treatment steps on MP retainment provided in section 1. On D3 and D4 the MP abundance at the outlet proved to be quite stable for both MP counts (D3: 1.2 ± 1.1 N/L, D4: 1.1 ± 0.6 N/L) and mass (D3: 6.7 ± 2.6 pg/L, D4: 7.8 ± 4.5 pg/L), but also comparable with that of D5 in terms of MP counts. Hence the release of MPs that occurred on D2 likely also involved the three following investigated days, whose MP counts abundance at the outlet was “sustained” with additional MPs. Since the MP mass abundance at the outlet on D3 and D4 was the highest recorded over the investigated period, some of the MPs were likely characterized by a larger size than on D2 (see also section 3.4).

Because of the persisting presence of MPs after the treatment, no significative difference (p > 0.05) could be seen for the MP counts and mass abundances between the inlet and outlet or among the investigated days. Note that Fig. 4 takes into account the overall MP abundances without distinguishing among the different polymeric types, hence the variation between MP counts and estimated mass should be considered indicative only.

The water retention time in the treatment works, including its storage tanks, was overall short, hence each sampling day was considered independently. Consequently, the retention time in the sand filters, despite being unknown, was assumed shorter than 24 h, provided that the plant operated continually.

A daily mean MP removal efficiency could hence be calculated from equation (2). The production of MPs on some days suggested that a release of a significant number of MPs may have occurred between the inlet and outlet. Overall, the accumulated efficiency at the treatment plant was 43.2 ± 45.9% in terms of counts, and 75.1 ± 28.2% in terms of mass, indicating that while retainment fluctuated from day to day, with some days even seeing a release of MPs, the drinking water treatment plant on average retained MPs. The difference between the two MP retainment efficiencies might be related to the breaking process involving the larger MPs from the inlet at the sand filtration step, which will cause the MP counts retainment to decrease, as opposed to the MP mass retainment. The treatment efficiency was furthermore comparable to those reported by previous studies [3].

The variabilities around means depicted in Fig. 4 were found from the three replicates which are behind each mean value. However, although the three replicates were taken in parallel from the same water, the MP distribution in the water flow may not have been homogeneous, hence a different number of particles may have reached the filter surfaces.

Further, a contributing factor to the variability can be the subsampling for the Raman analysis, where 25 out of 1000 μL were subsampled per replicate. When subsampling, it was assumed that MPs were homogeneously distributed in the liquid matrix of the treated samples and the MP abundance was calculated by way of proportions. However, MPs might not have been homogeneously distributed even though the sample was thoroughly mixed before collecting a subsample for deposition. For the MP mass estimation, further uncertainty may come from the assumption of an ellipsoid shape and the way the volume of a particle was calculated, as this approach generally ignores shape variations (despite the scarce presence of elongated morphotypes in the analyzed samples, see section 3.4). The standard deviation seemed generally related to the MP abundance value, hence D1 showed the highest MP counts and estimated mass as well as the largest error. Finally, it should be considered that Fig. 4 includes the identified MPs altogether, hence part of the standard deviation derived from the error associated with the abundance of the different polymer types over the investigated period.

Non-plastics were quantified for comparison: many of these would have been natural organic and inorganic particles, however, their materials were not identified (hence mass could not be calculated as non-plastics were not assigned a chemical ID). The removal efficiency of the non-plastic particles was 32.8 ± 0.9% based on particle counts. Since the removal efficiency of MPs was above that of non-plastics, one could expect the MP to non-plastic ratio at the plant's outlet to be lower than that of the inlet. However, this was not the case, suggesting again that MPs were created in the treatment works at least during some days. How this happened was not investigated but could be due to mechanical stress on plastic elements within the facility (e.g. valves, o-rings, sealing components, compressors, etc.) or the breakdown of larger MPs in the sand filter (further details are given in Supplementary Information).

Albeit there are many studies on MPs in drinking water in general, only one study on MPs in Danish drinking water has so far been published [42]. The authors sampled tap water in households and workplaces, analyzed particles down to 10 μm with μFTIR, and found an average abundance of 0.2 N/L for the 10–100 μm size range. The lower abundances found by Ref. [42] may have been due to the MP size limit of their study, which did not address the contribution of the 1–10 μm particles. Indeed, the spectral resolution limit of μFTIR does not make it suitable to analyze items much below 10 μm, as opposed to μRaman, which can go into the sub-micron size range. If the abundance at the outlet of a waterworks remains unchanged until the water reaches the tap, the abundance found in the present study was, apart from D1, higher than those found by Ref. [42].

Pitroff et al. [43] investigated the MP occurrence in drinking water produced from groundwater by two plants in Germany down to 5 μm using μRaman, finding 66 ± 76 N/m^3^ at the outlet. The authors employed a cascade filtration apparatus to sample particles down to 5 μm and calculated the LOQ of the method from field blanks with equation (4). However, they found no significant difference between their drinking water samples and their blanks. Bäuerlein et al. [44] studied two drinking water plants fed from underground aquifers in the Netherlands and two household taps connected to the same network. LDIR (Laser Direct InfraRed) was employed to identify particles down to 20 μm, and in one of the plants the MP abundance at the inlet was 2223 N/m^3^. At one of the investigated taps, high levels of PA were detected, which the authors could not explain. Mintenig et al. [25] investigated 5 drinking water plants and related households in a German region served by treated groundwater. After sampling at different points in each facility (300–1000 L per sample) and at the taps (1200–2500 L per sample), μFTIR was employed for the MP analysis down to 20 μm. The overall average MP counts were 0.7 N/m^3^, and the authors speculated that abrasion of plastic equipment during the water treatment could be a source of MP. These examples of other studies show that direct comparison among studies can be challenging. Nevertheless, they also indicate that MPs occur in drinking water at roughly comparable concentrations as in the present studies, especially when allowing for lower size detection limits.

### 3.3 Polymer composition

**Fig. 5:**
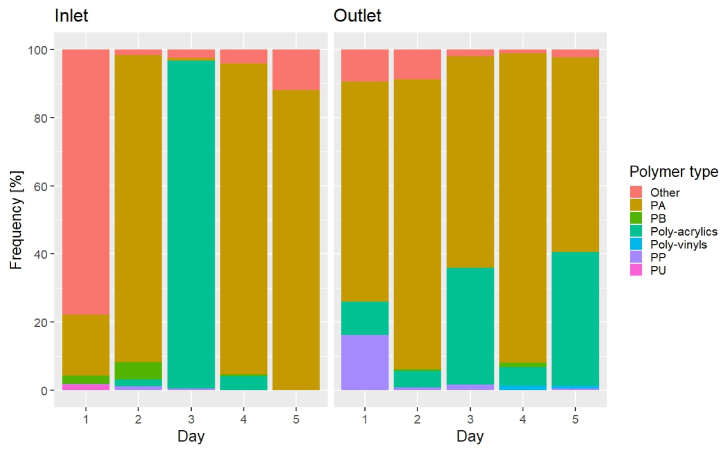
MP counts abundance as polymer frequency over time at the plant's inlet and outlet.

**Fig. 6:**
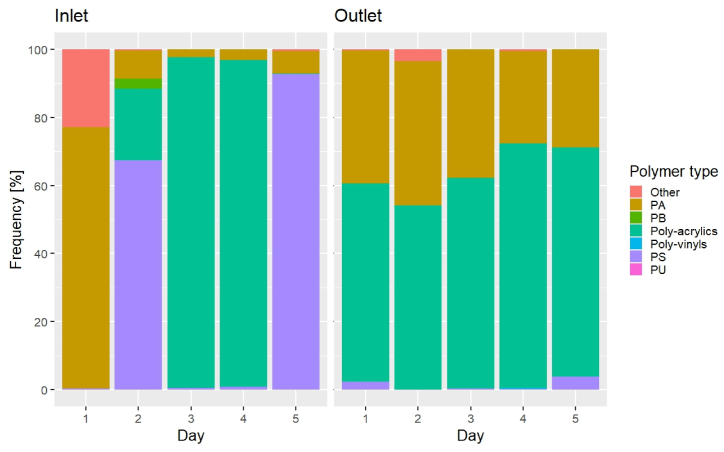
MP mass abundance as polymer frequency over time at the plant's inlet and outlet.

The polymer composition of the MPs might give further hints at how additional MPs were formed in the plant (Fig. 5, Fig. 6). In terms of counts, poly (amide) (PA) MPs dominated in 3 out of 5 inlet samples (mean 39%) and all outlet samples (mean 78%) (p < 0.05). The category “Other”, a mix of many different polymer types occurring in small numbers, dominated in the inlet on D1 (78%), while poly-acrylics dominated on D3 (96%). With respect to the mass estimate (Fig. 6), PA was the most abundant polymer in the inlet on D1 (77%), while it was only present at low concentrations on the other days. Instead, PS prevailed on D2 (67%) and D5 (93%), while poly-acrylics dominated on D3 and D4 (D3: 97%, D4: 96%). The outlet, on the other hand, showed a quite stable polymer composition, with poly-acrylics mass accounting for 55–72% (mean 65%) on all days, followed by PA with 27–42% (mean 34%). The origin of the MPs measured in inlet and outlet cannot be traced back, but there may well be plastic parts upstream of the sampling points consisting of PA and poly-acrylics, for example sealings in valves and fittings (notice the pure oxygen compressors before the inlet sampling point in Fig. S1). Here it should be kept in mind that the overall abundance in terms of MP mass was quite low, namely around 16 pg/L on average in the inlet to the sand filters (corresponding to 8 mg/year of plastic), hence a quite small amount of what totally could be present as part of the plant's construction. Further details are given in Supplementary Materials.

PA and poly-acrylics dominated the outlet, but were of different relative sizes, with MPs of PA being smaller than MPs of poly-acrylic. There was furthermore an overall retainment of all MPs, probably in the sand filters of the plant (Fig. S1). It seems reasonable to assume that the sand filters smoothed out the MP load, retaining preferentially the larger ones, but also releasing the smaller ones.

### 3.4 Microplastic morphological analysis

**Fig. 7:**
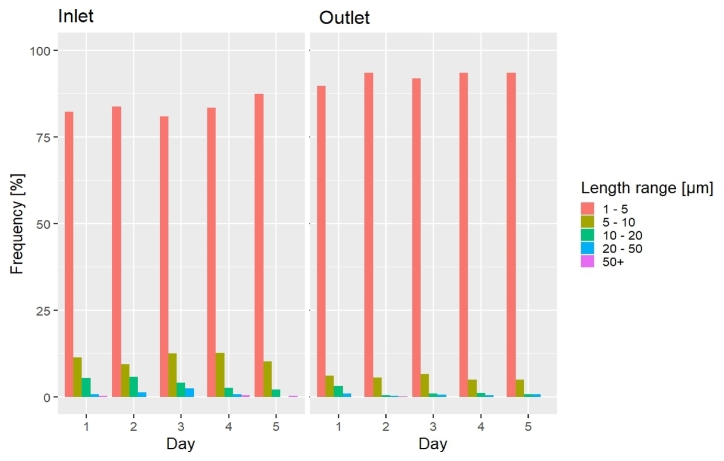
MP length ranges frequency over Days 1–5 at the plant's inlet and outlet.

The data behind the morphological analysis were not blank corrected. The largest MP particle found in the samples was 204.6 μm long and the MP median length was 2.4 μm. Fig. 7 shows MPs divided into five size classes: 1–5 μm, 5–10 μm, 10–20 μm, 20–50 μm, and 50+ μm in length. In all samples, the most populated size class was 1–5 μm (mean inlet 83.3%, mean outlet 92.7%, p < 0.05), followed by the 5–10 μm class (mean inlet 11.9%, mean outlet 5.7%). The frequencies of the larger size classes were far lower than the 1–5 μm and 5–10 μm ones. The 10–20 μm class held 3.6% and 1.0% of the mean inlet and outlet, respectively, while the 20–50 μm class held even less (mean inlet 0.9%, mean outlet 0.6%), and the 50+ μm class held the least number of MPs (mean inlet 0.3%, mean outlet <0.1%). Interestingly, on D3 and D4 the 10–20 μm frequency at the outlet was higher than on D2 and D5 (0.94 and 1.09% respectively), which could explain the related higher MP mass abundance in Fig. 4. Similarly, the 20–50 μm frequency showed a slight increase on D3 and D4 at the outlet if compared with D2, and it was comparable with that of D5. The abundance of the 1–5 μm particles in the inlet was 28.56 N/L while the outlet held 16.83 N/L, i.e., only 41.1% of this size class was on average retained. For 5–10 μm MP, the abundance was 4.06 N/L and 1.03 N/L, respectively, i.e., 74.6% were on average retained. For MPs >10 μm, numbers were significantly lower, but retainment was significantly higher, namely 0.55 N/L in the inlet, 0.099 N/L in the outlet, and hence 81.3% retainment. Therefore, the sand filters seemed to preferentially retain the MPs above roughly 5–10 μm [45], possibly breaking the larger MPs into smaller particles Also, larger MPs may have been retained by sedimentation in the storage tanks before the treated water was sampled, which is a process mostly depending on particle size, roughness, and density [46]. Consequently, the frequency of the 1–5 μm MP at the outlet overall increased.

The findings confirmed the common observation that there tend to be more small MPs than large ones, for example [47,48], who also employed μRaman in their studies. Ref. [39] however found that only 32% of MPs were smaller than 20 μm compared to 98.8% (inlet) and 99.4% (outlet) found in the present study. This might well be due to differences in analytical techniques, as they used μFTIR in transmission mode, where the absorption signal decreases with the particle thickness. This problem does not occur for μRaman, which is a surface analysis technique. Classifying MPs with a ratio between length (Feret maximum diameter) and width (Feret minimum diameter) ≥3 as fibres and the rest as fragments [2] showed that fibre counts in the inlet were higher than in the outlet on D1, D4, and D5, while it was the other way around on D2 and D3. Hence there was no systematic trend to fibres being more or less retained compared to fragments when measured as MP counts. Overall, fragments were the most common shape, accounting for more than 98% of the MPs. This stands in contrast to what Kanganike and Babel [49] found in their study on MPs in drinking water during the rainy and dry seasons in Thailand. Their study focused on a drinking water plant fed by a river and applying clarification, dual filtration with sand and anthracite coal, and chlorination. The authors speculated that the prevalence of fibres in the samples (30–40%) was due to the relatively high MP abundance in the river used to feed the investigated waterworks, and to the inherent ability of the fibres to escape filter pores.

### 3.5 Nanoplastic qualitative exploration

**Fig. 8:**
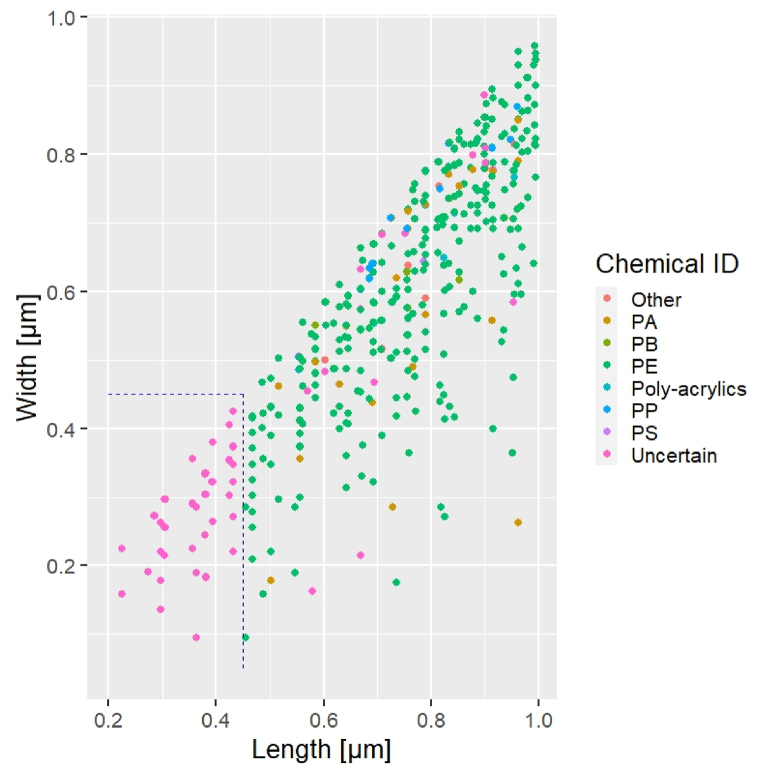
Length vs width (Feret maximum vs Feret minimum diameters) for all analyzed NPs. The blue dotted line indicates the size limit above which the NP spectra presented a HQ above 50%. (For interpretation of the references to colour in this figure legend, the reader is referred to the Web version of this article.)

While analyzing the drinking water samples, several sub-micron particles were observed in the visible images taken in dark field, i.e., particles of smaller size than the nominal pore size of the filter employed in the sampling and sample preparation. A subset of these was analyzed after filtering out particles above 1 μm in length and width (Fig. 1). Three sub-areas of 200 × 200 μm were selected from each deposition area and analyzed, yielding a total of 621 sub-micron particles with plastic-like Raman spectra. As previously stated, a poorer HQ (50%) was accepted for the NP evaluation compared to the MP analysis (80%), yielding lower confidence in the NP versus MP material assignment. Only NPs having both dimensions above 0.45 μm could be considered, as the HQ for smaller particles was below 50% when compared to the library references (area delimited by the blue dotted line in Fig. 8). Furthermore, analyzing single items below 0.45 μm would be technically demanding due to the Airy diffraction limit, which is ∼0.36 μm with a 532 nm laser and a 100 × objective.

No quantitative results could be drawn from the NP evaluation since the filters used for sampling and sample preparation had a nominal pore size of 1 μm and the field blanks were not analyzed for the NPs. However, this evaluation illustrates the potential and limitations of μRaman for NP analysis of environmental samples and shows that the development of reliable analytical protocols for these analytes deserves further research. In comparison with other known categories of nanopollutants, NP quantification and interaction with living organisms are still considered an open challenge [50], and μRaman seems able to push the analysis for plastic particles into the nanoscale. The work of Sobhani et al. [51] showed that characteristic Raman signals of environmental particles down to 100 nm can be experimentally obtained, even though with decreasing spectral quality as smaller particles were considered. Moreover, Frehland et al. [52] demonstrated that the removal rates of the NPs (66.5%) and MP fibres were correlated to that of the total suspended solids in a pilot wastewater treatment plant. As suggested by the outcomes of the present study regarding MPs in a drinking water plant, contributions from plastic parts within such a plant may occur, and the sand filters typically applied probably will be only partly effective to retain NPs.

### 3.6 Human microplastic intake

The WHO Guidelines for Drinking – water quality (WHO 2009) [53] recommended an average adult drinking water consumption of 3 L/(day·capita). Applying this intake and the average MP abundance in the outlet of the treatment plant (1.4 N/L of MPs >1 μm), an annual adult MP intake *via* tap water can be estimated as 1533 N (4.4 ng/(year·capita)). The MP count abundance is several orders of magnitudes above the estimation by Ref. [39], and the main reason for the difference is that the present study achieved a lower size quantification limit. To make a comparison with other major exposure pathways, the review of Bai et al. [4] reported a total dietary intake of 1.42–1.54 ·10^5^ N/(year·capita), while [2] calculated an average inhaled intake of 272 N/h for a male individual (2.38·10^6^ N/(year·capita)). Hence drinking water produced from a protected groundwater source would account for only a small part of the potential total MP intake by humans. However, human MP intake estimations should generally be taken with care, as much uncertainty is still associated with MP exposure routes, absorption, and excretion rates, and finally, toxicity [54].

## 4 Conclusions

The studied drinking water plant was able to retain MPs (i.e., plastic particles >1 μm), although to a different extent depending on the MP size. The water in its outlet held 1.4 N/L, corresponding to an estimated MP mass of 4 pg/L. It achieved an average removal over 5 days of 43.2% in terms of MP counts. The efficiency was best for the larger MPs, deteriorating as particles became smaller than approx. 5–10 μm. Below 5 μm the efficiency was as low as 41.1%, while it increased to 74.6% for the 5–10 μm range, and to 81.3% for MPs >10 μm. The efficiency for MP mass retainment was higher, accounting for 75.1%. Extrapolating measured MP masses to a full year of operation showed that approx. 8 mg/year of plastic entered the sand filters, which is sufficiently low to allow the assumption that the MPs could have originated from the aging of plastic parts upstream of the filters.

The retainment efficiency of the facility was highly variable over the investigated period. This dynamic behavior means that substantial uncertainty is related to single measurements and assessing the operation of such a plant hence requires intensive monitoring. The preferential retainment of larger MPs compared to the smaller ones furthermore show that a measured retainment efficiency will depend heavily on the lower size quantification limit, and hence the analytical approach. This also impacts the interpretation of human exposure from the intake of MPs from drinking water. Finally, the study showed that NPs were present in the analyzed drinking water samples, and that μRaman was able to characterize at least the larger ones.

## Supplementary Information

Supplementary Information: μRaman spectra for the identified polymer, additional experimental details and methods, including photographs of experimental setup.

## Author contribution statement

Luca Maurizi 1) conceived and designed the experiments; 2) performed the experiments; 3) analyzed and interpreted the data; 4) contributed reagents, materials, analysis tools or data; 5) wrote the paper.

Lucian Iordachescu 2) performed the experiments; 4) contributed reagents, materials, analysis tools or data; 5) wrote the paper.

Inga V. Kirstein 3) analyzed and interpreted the data; 5) wrote the paper.

Asbjørn H. Nielsen 3) analyzed and interpreted the data; 5) wrote the paper.

Jes Vollertsen 3) analyzed and interpreted the data; 5) wrote the paper.

## Data availability statement

Data will be made available on request.

## Additional information

Supplementary content related to this article has been published online at [URL].

## Declaration of competing interest

The authors declare that they have no known competing financial interests or personal relationships that could have appeared to influence the work reported in this paper.
