# Supplementary material for: Do drinking water plants retain microplastics? An exploratory study using Raman micro-spectroscopy
Source: Heliyon. 2023 Jun 9;9(6):e17113. doi: 10.1016/j.heliyon.2023.e17113 (PMC10361326; doi:10.1016/j.heliyon.2023.e17113)
Supplement: Multimedia component 1 [file mmc1.docx]

Do drinking water plants retain microplastics? An exploratory study using Raman micro-spectroscopy

*Luca Maurizi, Lucian Iordachescu, Inga V. Kirstein, Asbjørn H. Nielsen, Jes Vollertsen*

**Supplementary Information**

# Drinking water sampling and sample preparation protocol

Figure S1 is a scheme of the drinking water plant.


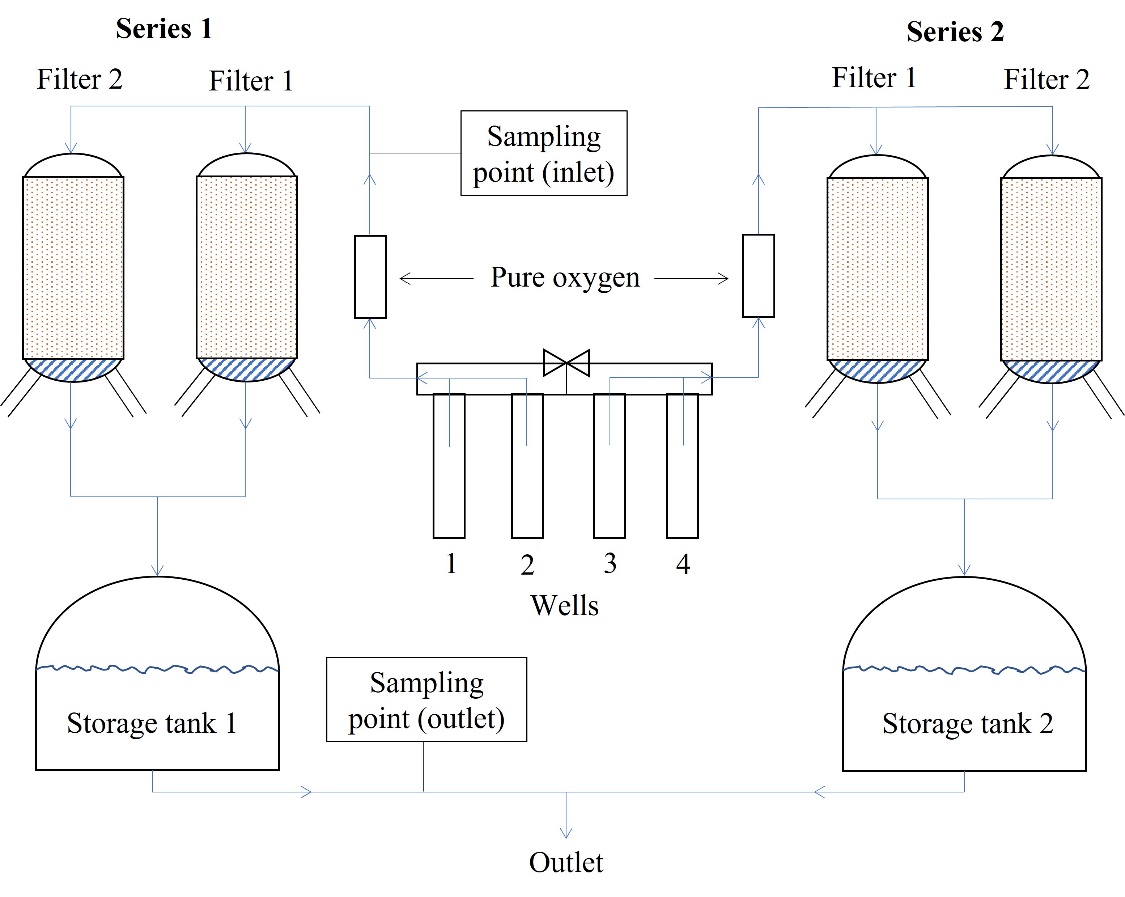


Figure S1. Scheme of the investigated drinking water plant (not in scale).

The employed sampling device (Figure S2) was developed and built at Aalborg University (Denmark). It is made of type 316 stainless steel, and it is constituted of four flow lines, along which filter holders can be connected in series. The device’s inlet can be connected to a water network through a flexible pipe, while the outlet of each flow line can be equipped with a flowmeter to check the volume being sampled.


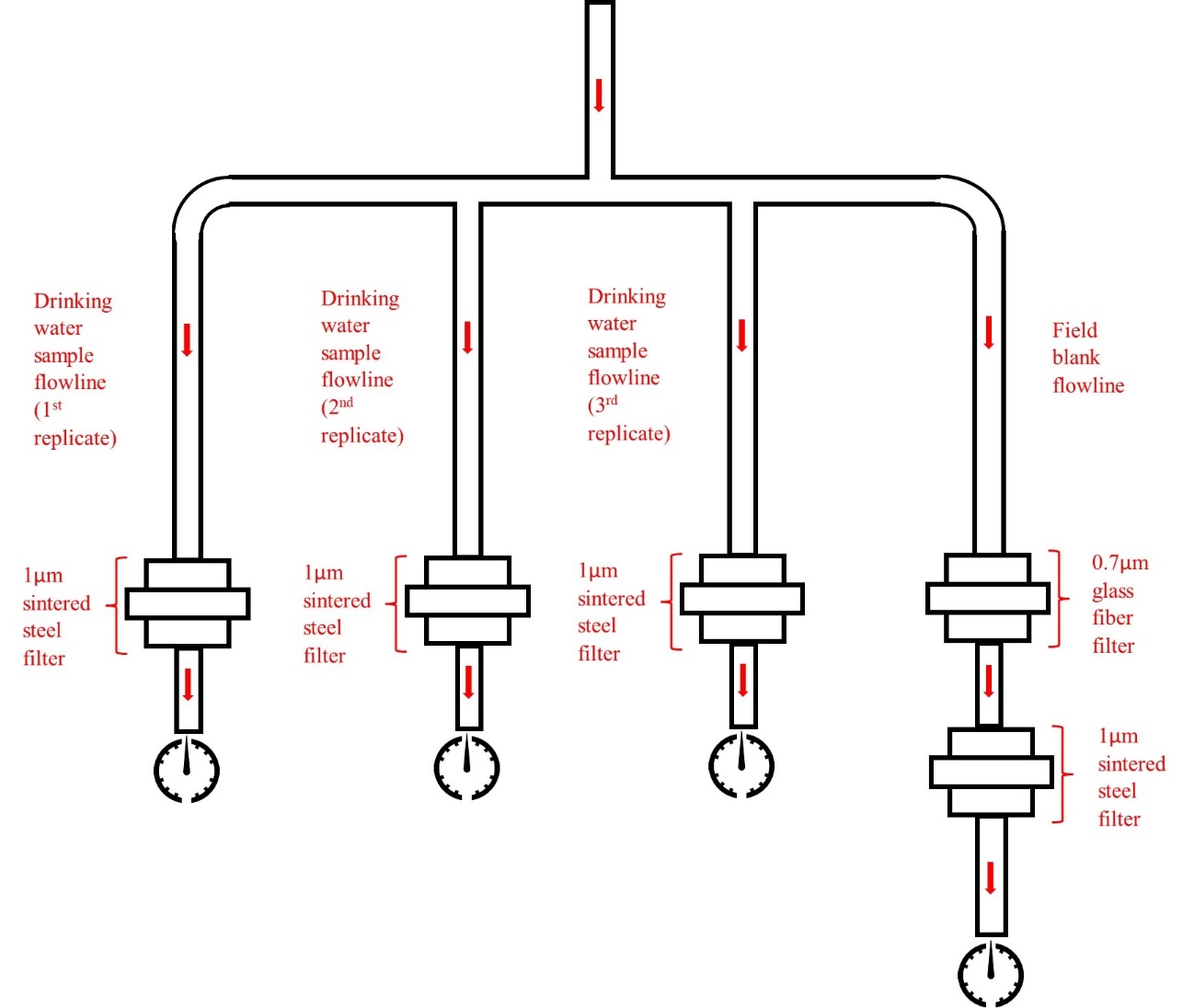

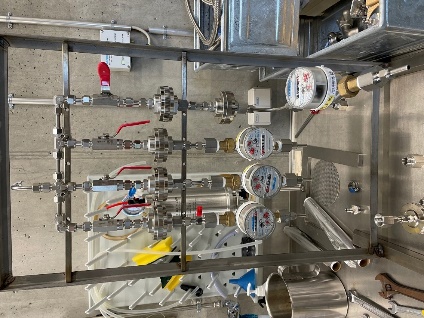


*Figure S2. Aalborg University drinking water sampling device (left) and its simplified scheme (right).*

Figure S3 is a scheme of the protocol followed to treat the drinking water samples.

*Figure S3. Scheme of the sample preparation protocol.*


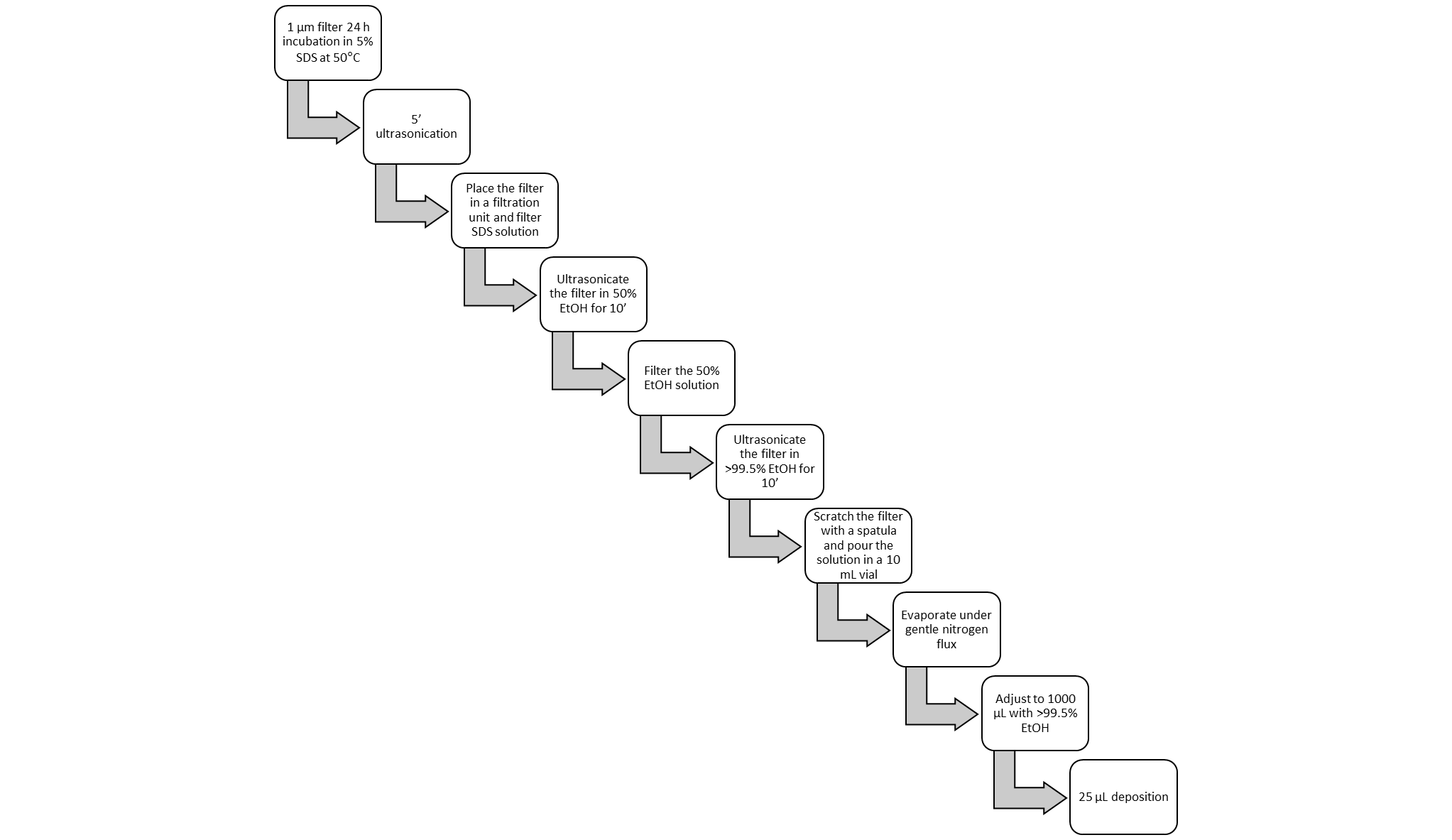


Each filter was incubated in 5% Sodium Dodecylsulphate (SDS, VWR, Germany) at 50°C for 24 hours inside a beaker in a thermal bath (Thermo Fisher Scientific, Germany). This step was aimed at removing the organic matter adhering to the environmental MP and NP surfaces, which can produce fluorescence when performing the µRaman analysis. After sonicating the filter in the 5% SDS solution for 5 minutes, the SDS was filtered through the same filter in a glass filtering system connected to a vacuum pump (VWR, Germany). Then, the filtering system was dismounted and the glass parts in contact with the filter were thoroughly rinsed with 50% ethanol (EtOH for HPLC, Th. Geyer GmbH, Germany), letting the 50% EtOH solution percolate into a new beaker. The filter was removed and put into the same beaker with the particle-enriched 50% EtOH solution. More 50% EtOH was added until the filter was fully immersed in a layer of ethanolic solution. The beaker was covered with aluminum foil to prevent external contamination and ultra-sonicated for 10 minutes (Elmasonic S40H, ABC Clean, Denmark) to dissolve the SDS in the ethanolic solution. A new glass filtering unit was equipped with the same filter, and the particle-enriched 50% EtOH solution was filtered through it. The filter was put into a new beaker and the system’s glass parts were rinsed with >99.5% EtOH, pouring the solution into the beaker with the filter. Once the filter was immersed in a layer of >99.5% EtOH, the beaker was covered with aluminum foil and ultra-sonicated for 10 minutes. Finally, the filter was scratched with a clean steel spatula on both sides and rinsed with a small amount of >99.5% EtOH to maximize particle recovery. After removing the filter from the beaker, the particle-enriched >99.5% EtOH solution was poured into a clean 10 mL glass vial and kept under gentle nitrogen flow in an evaporator at 55°C (TurboVap Biotage, Sweden) until exhaustion of the entire volume. The dried vials were kept in the fridge at 5°C until further processing and analysis.

# Deposition for Raman analysis

Figure S4 is an example of reconstituted sample ready for deposition. The particle-enriched mixtures looked clear, and no precipitate could be seen.


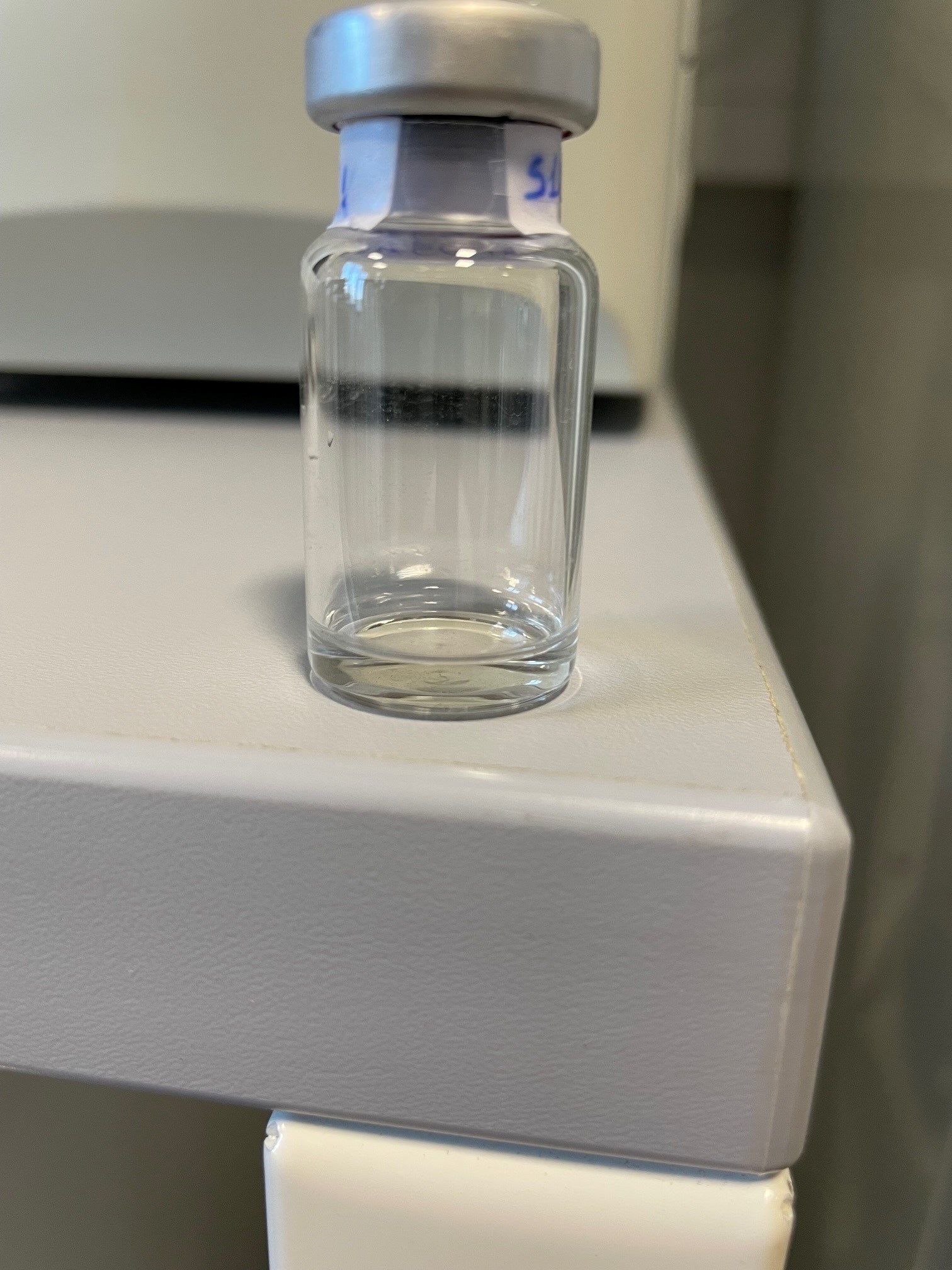


Figure S4. Example of reconstituted sample in a 10 mL vial. The mixtures were clear, and no precipitation of particles could be observed.

Sample deposition was performed through a device (Figure S5) developed at Aalborg University. It consists of the following components:

1. Metallic main body with a lateral aperture and a circular hole in the upper side
2. Microscope glass slide
3. Metallic funnel with 2 mm hole
4. 10 × 10 mm Silicon substrate

By inserting the glass slide in the aperture on the left, the Silicon substrate may be put onto it in the center of the main body’s hole. The metallic funnel may then be placed onto the Silicon substrate so that the particles deposed from the sample are effectively concentrated on the substrate within an area of about 2 mm diameter.


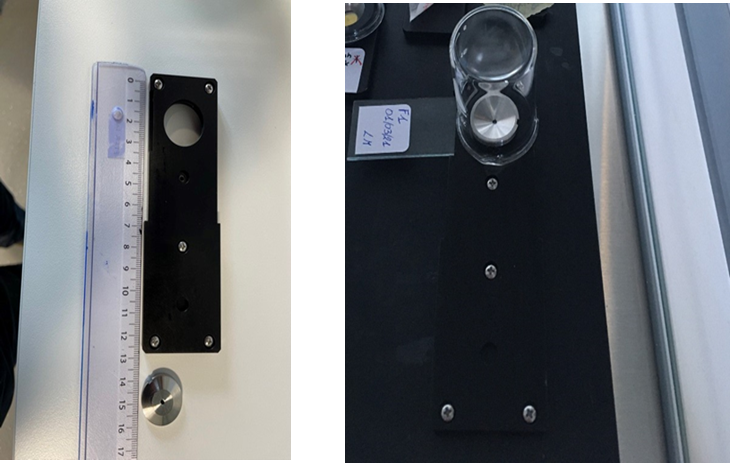


a) b)

Figure S5. The deposition device for Raman analysis developed at Aalborg University. a) Particular of the main metallic body and 2 mm-hole funnel; b) A sample drying after deposition.

Figure S6 is the Raman spectrum of the crystalline Silicon employed as a substrate for sample deposition. Two peaks can be clearly distinguished: the first-order Raman scattering at 519 cm^-1^ and the second-order transition (overtone) at about 950 cm^-1^. These two signals could be easily subtracted from the particles’ raw Raman spectra.


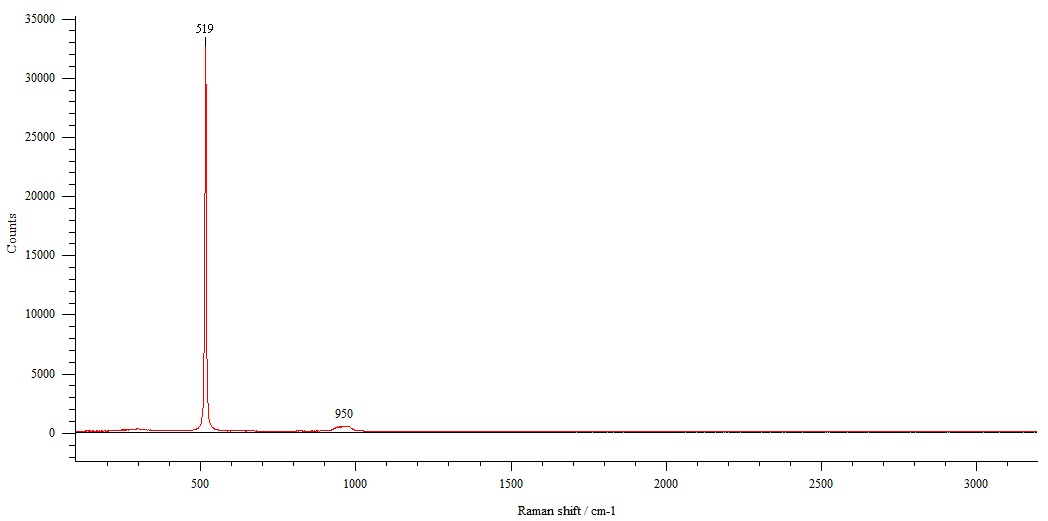

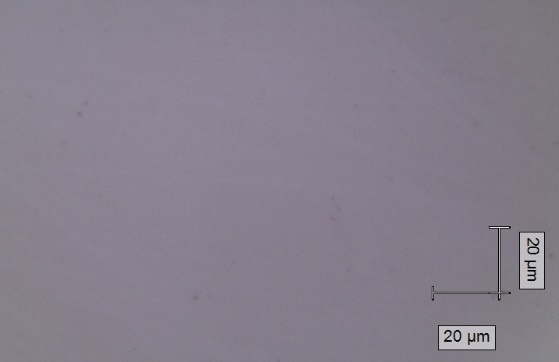


Figure S6. Single-crystal Silicon substrate Raman spectrum with visible image at 50× (right). The spectrum was taken at 50× with a 488 nm laser. The peak at 519 cm^-1^ represents the first-order Raman scattering of Silicon crystals, while the peak at ~950 cm^-1^ indicates the second-order (overtone) Raman scattering.

# Active area after deposition and µRaman analysis

Figure S7 is an example of a deposed active area from one of the analysed samples. The three white squares indicate the points considered for the NP qualitative investigation. For the MP quantification, instead, the entire circular area was analysed.


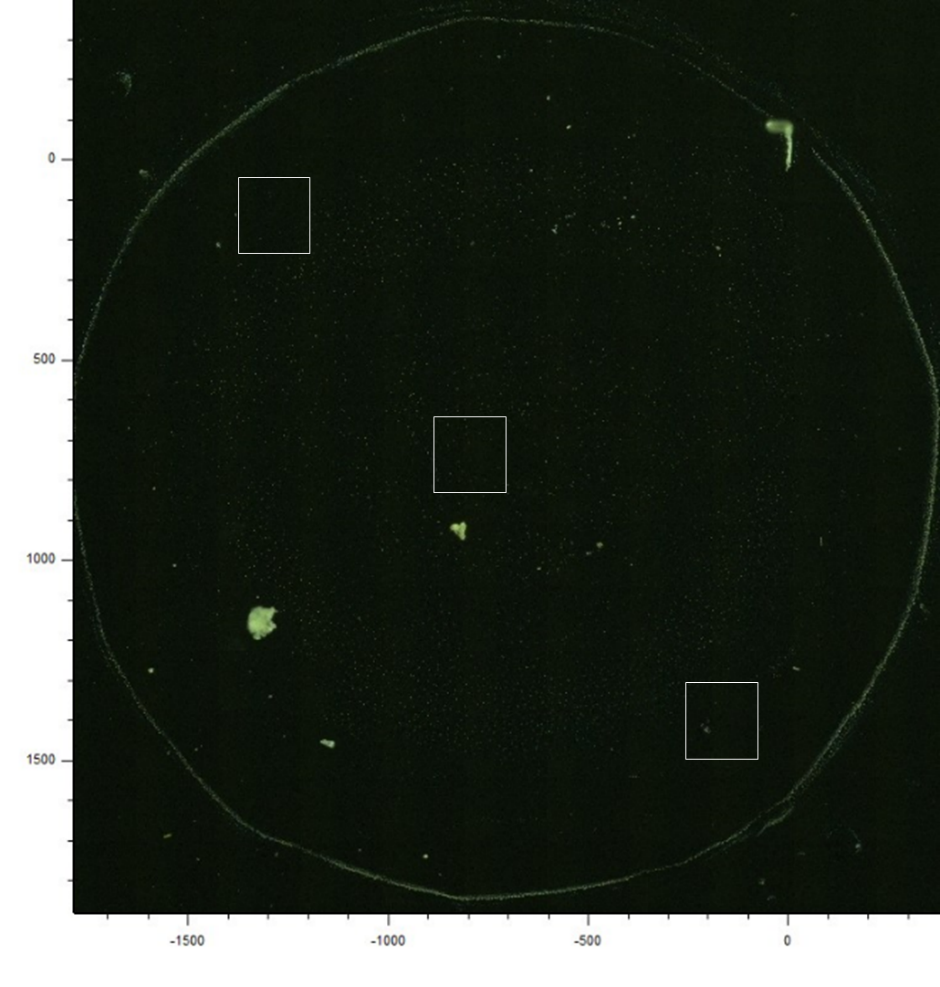


Figure S7. Example of a deposed area from one of the analysed drinking water samples (DF, 50×).

Before the spectral acquisition, a visible montage of the area to be analysed was taken in DF (Dark Field) mode:

- MP analysis of the field blanks, drinking water samples, and recovery samples: the entire circular area (approximately 3.14·10^6^ µm^2^) obtained by deposition was considered (Figure S7);
- Exploration of the NP fraction in the drinking water samples: three sub-areas of 200×200 µm (4·10^4^ µm^2^) were selected within the circular area (Figure S7), in positions corresponding to the top-left, center, and bottom-right.

The visible images were analysed with the Renishaw software ‘Particle Analysis’. The first parameter to be set was the Intensity, which is the visible light intensity threshold to be reached by an object in the image, in order to be added to the waiting list of the software. The Intensity value was manually set to exclude possible random features of the Si substrate (e.g. scratches), which could be counted by the software as false positives, and the same Intensity was then adopted for all the samples. At this stage, the software also calculated the morphological features of each object on the waiting list. Secondly, the objects to be analysed could be virtually filtered by setting different thresholds for the morphological parameters "Length" and "Width". For the MP analysis of the field blanks, drinking water samples, and recovery samples, length and width were set to >1 µm (i.e., all objects with major or minor dimensions below 1 µm were excluded), whilst for the NP analysis of the drinking water samples, length and width were set to <1 µm. Finally, the laser was automatically driven by the software onto each selected object on the waiting list, and its Raman spectrum was acquired according to the chosen analysis parameters.

Figures S8 – S12 are examples of visible images of MPs and NPs taken at 50× or 100× magnification in BF and DF. It could be seen that the MPs were transparent and of different shapes (see also Supplementary Information, "5. Morphological analysis").


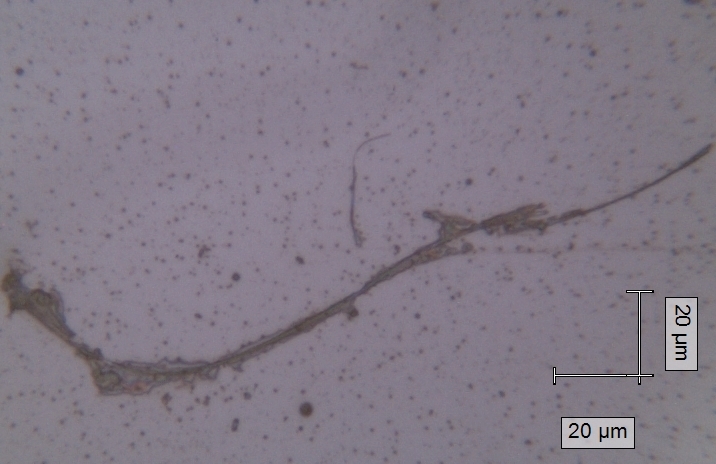

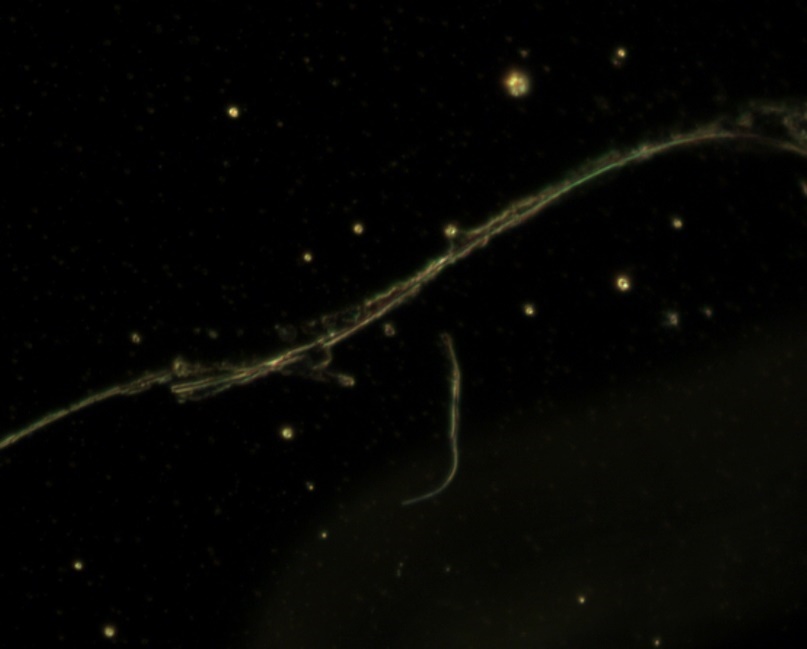


Figure S8. Picture of PA fibre at 50× in a) BF and b) DF.

*a) b)*


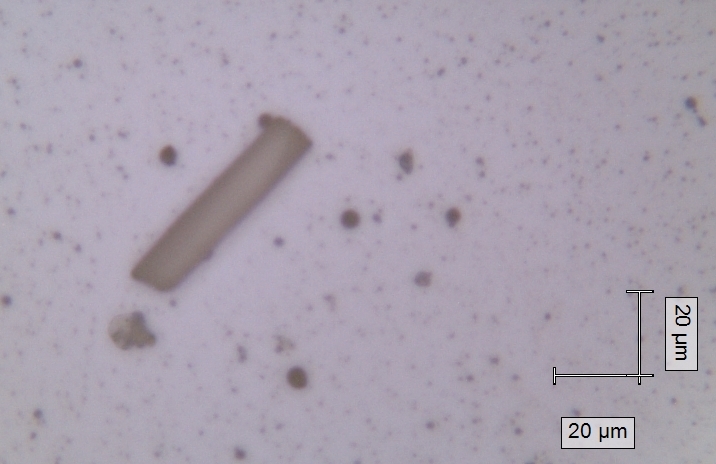

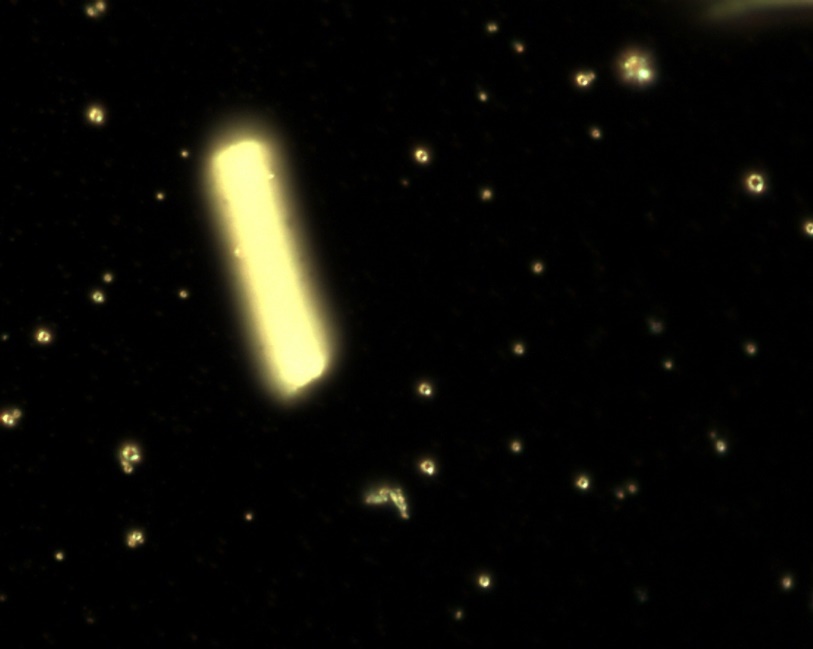


1. *b)*

Figure S9. Picture of cylindrical poly-acrylic MP at 50× in a) BF and b) DF.


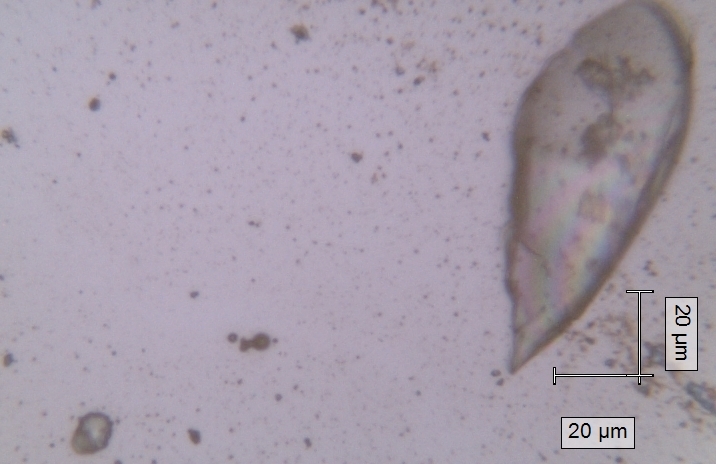

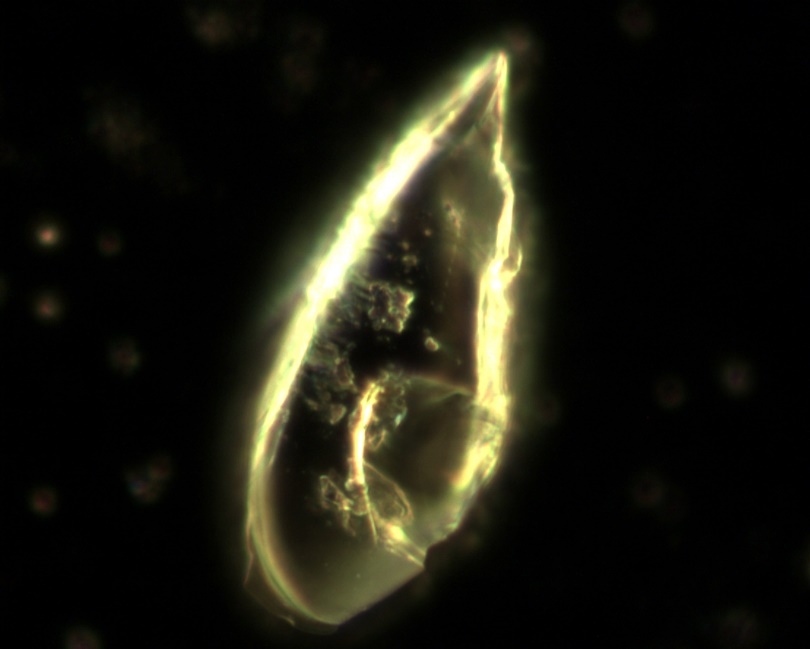


*a)*  *b)*

Figure S10. Picture of PS fragment at 50× in a) BF and b) DF.

a) b)


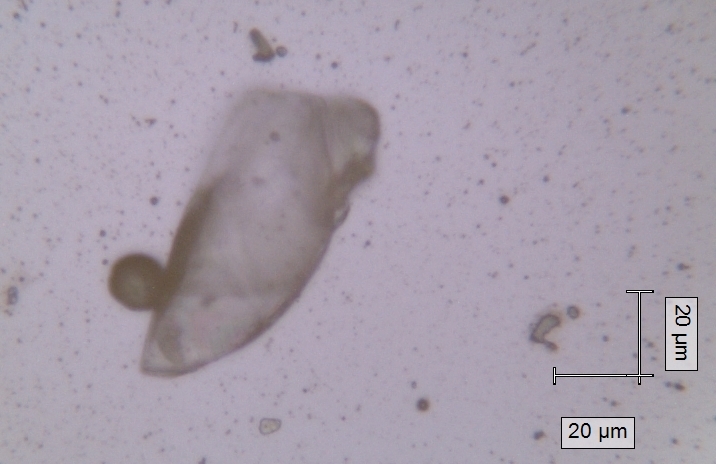

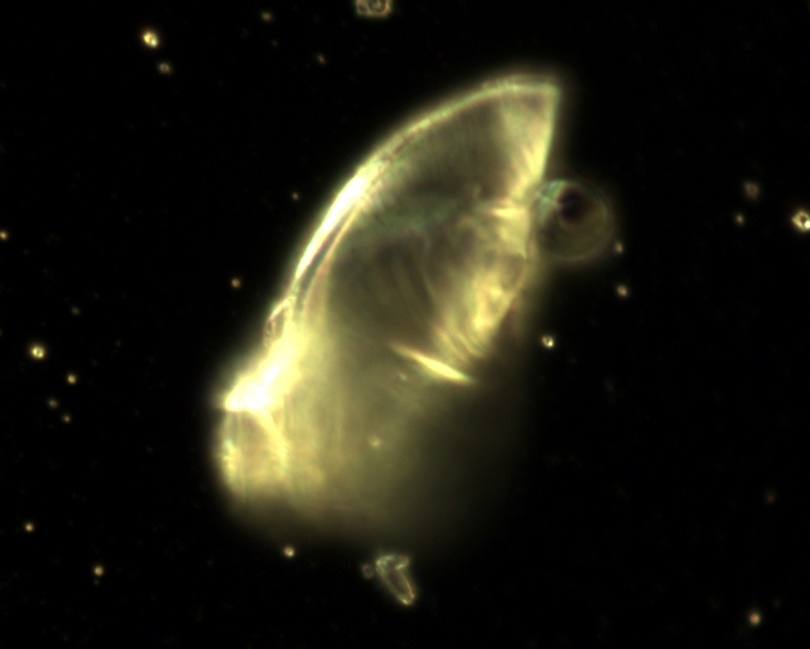


Figure S11. Picture of PP fragment near a spherical PU MP at 50× in a) BF and b) DF.


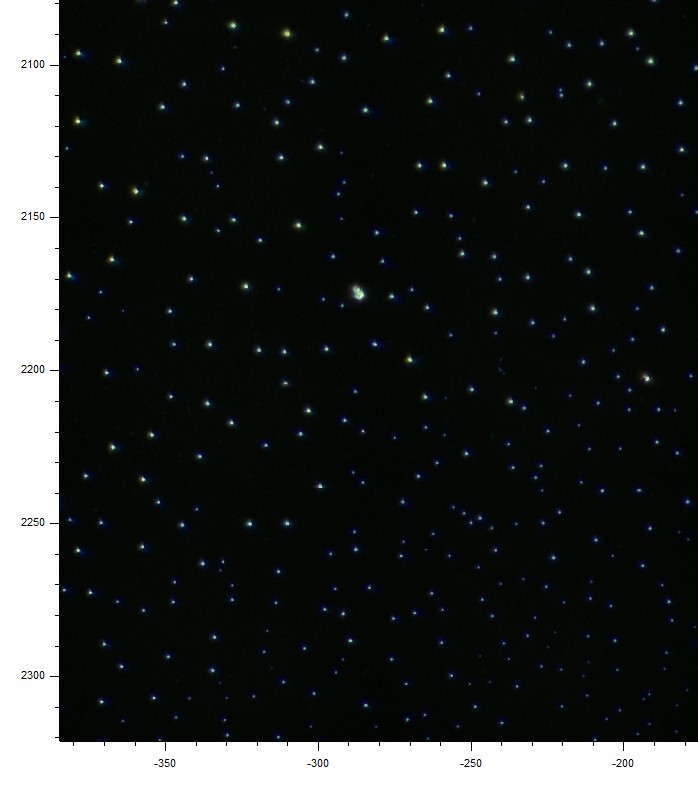


Figure S12. Example of 200×200 µm sub-area (DF, 100×).

# Microplastic quantification

For simplicity and graphical readability, the identified plastic types were grouped into the following categories:

- Other: (poly(ethylene glycol), poly(acenaphthylene), poly(caprolactone triol), poly(1,4cyclohexanedimethylene terephthalate-co-ethylene terephthalate), poly(isoprene), poly(oxazoline), poly(norbornene), poly(methyl-methacrylate), and techtron PPS)
- PA: (nylon 11, nylon 12, nylon 6, nylon 6(3)T, nylon 6,10, nylon 6,12, nylon 6,9 and poly(amide) resin)
- PB: (poly(butadiene), poly(butyl acrylate), poly(butyl terephthalate), and poly(butyl terephthalate ester))
- Poly-acrylics: (poly(acrylic acid), poly(acrylamide), poly(acrylamide carboxyl), poly(acrylamide-co-acrylic acid), poly(acrylonitrile))
- Poly-vinyls: (poly(vinylacetate), poly(vinylalcohol), and poly(vinyl stearate))
- PP: (poly(propylene), poly(propylene chloride), and poly(propylene-co-butene))
- PS: (poly(styrene), poly(acrylonitrile-butadiene-styrene), poly(styrene-butadiene), and poly(stryrene-isoprene))
- PU: (poly(urethane))

## MP quantification in the field blanks

- MP counts: 286 ± 263 N. Polymeric distribution: nylon 12 8.4%, nylon 6 7.7%, nylon 6,10 16.0%, PA 8.2%, PEG 5.4%, PET 6.5%, poly(acrylamide) 5.1%, poly(acrylamide-co-acrylic acid) 5.0%, poly(dimethyl siloxane) 8.5%, poly(propylene) 4.5%, poly(styrene) 13.6%, and PVC 11.1%.
- MP mass estimate: 2432.21 ± 5863.23 pg. Polymeric distribution: nylon 12 0.3%, nylon 6 11.7%, nylon 6.10 1.4%, PA 9.9%, PEG 0.1%, PET 58.4%, poly(acrylamide) 0.7%, poly(acrylamide-co-acrylic acid) 10.8%, poly(dimethyl siloxane) 1.6%, poly(propylene) 0.9%, poly(styrene) 2.0%, and PVC 2.1%.

## MP counts recovery

Our choice to employ 3 different standard polymers was an attempt to replicate the variety of plastic polymers usually populating real drinking water samples, even though the usage of environmental (i.e. degraded) MPs may lead to a different outcome for the recovery assessment.

Generally, the result of a recovery test is subject to the contributions of all the stages composing an analytical method for MP analysis: in the current case, sampling, sample preparation, analysis, and recovery rate estimation. During the sampling in the field, MPs may have been lost due to insufficient water tightness between the o-rings and the filters in the filter holders. To take into consideration this possibility, the same sampling protocol as for the field samples was employed for the positive controls, by filtering approximately 1 m^3^ of tap water (see also section 2.2 "Preparation of recovery samples").

The sample preparation was another contributor to the particle loss, which could be minimized by frequently rinsing the glassware employed and limiting the number of steps as far as possible. However, the glassware was judged recovered by visual inspection, and it was assumed the steel filters be completely recovered at the end of the sample preparation (see also section 2.4 "Sample preparation and analysis").

When setting the Intensity value in the "Particle Analysis" software, the aim was to include as many particles as possible from the sample’s active area. This could have been achieved by setting the brightness threshold to a low value, which would also have resulted in a waiting list potentially populated by a high number of false positives (e.g. scratches or features of the substrate). Setting the brightness was, therefore, a matter subject to a compromise between analytical accuracy and analytical run-time. No reduced-area patterns were applied during the MP analysis to avoid adding further sources of error ([1], [2]). Finally, another source of uncertainty came from the usage of proportion when calculating the recovery rate Q (see also section 2.5 "Post-processing of the µRaman data"). It was assumed that the standard MPs were homogenously distributed inside the liquid matrix of the recovery samples, which does not seem to reflect the experimental reality.

## MP quantification in the drinking water samples

Table S1 summarizes the overall MP counts (N/L) and estimated mass (pg/L) for each investigated day at the plant’s inlet and outlet.

| **Sample** | **MP abundance [N/L]** | **MP abundance [pg/L]** |
| --- | --- | --- |
| D1i | 6.3 ± 11.7 | 31.8 ± 56.3 |
| D1o | 0.1 ± 0.1 | 0.6 ± 0.6 |
| D2i | 1.0 ± 0.6 | 14.9 ± 29.5 |
| D2o | 3.9 ± 5.6 | 2.2 ± 1.7 |
| D3i | 2.1 ± 0.9 | 10.6 ± 15.0 |
| D3o | 1.2 ± 1.1 | 6.7 ± 2.6 |
| D4i | 2.3 ± 1.9 | 16.2 ± 31.4 |
| D4o | 1.1 ± 0.6 | 7.8 ± 4.5 |
| D5i | 0.8 ± 0.4 | 6.5 ± 7.9 |
| D5o | 0.8 ± 0.5 | 2.6 ± 1.5 |
| **Mean inlet** | 2.5 ± 2.0 | 16.0 ± 8.5 |
| **Mean outlet** | 1.4 ± 1.3 | 4.0 ± 2.7 |

Table S1. Overall MP counts (N/L) and estimated mass (pg/L) at the plant’s inlet and outlet for each investigated day.

Table S2 reports on the MP removal efficiency over the five investigated days.

| **Day** | **Counts removal efficiency [%]** | **Mass removal efficiency [%]** |
| --- | --- | --- |
| 1 | 98.4 ± 1.7 | 98.0 ± 1.8 |
| 1+2 | 45.2 ± 1.4 | 94.0 ± 10.0 |
| 1+2+3 | 44.7 ± 45.0 | 83.4 ± 20.9 |
| 1+2+3+4 | 46.1 ± 44.7 | 76.5 ± 24.1 |
| 1+2+3+4+5 | 43.2 ± 45.9 | 75.1 ± 28.2 |

Table S2. MP removal efficiency over the five investigated days with mean values.

Figure S13 shows the MP/non-plastic ratio over the investigated period at the plant’s inlet and outlet.


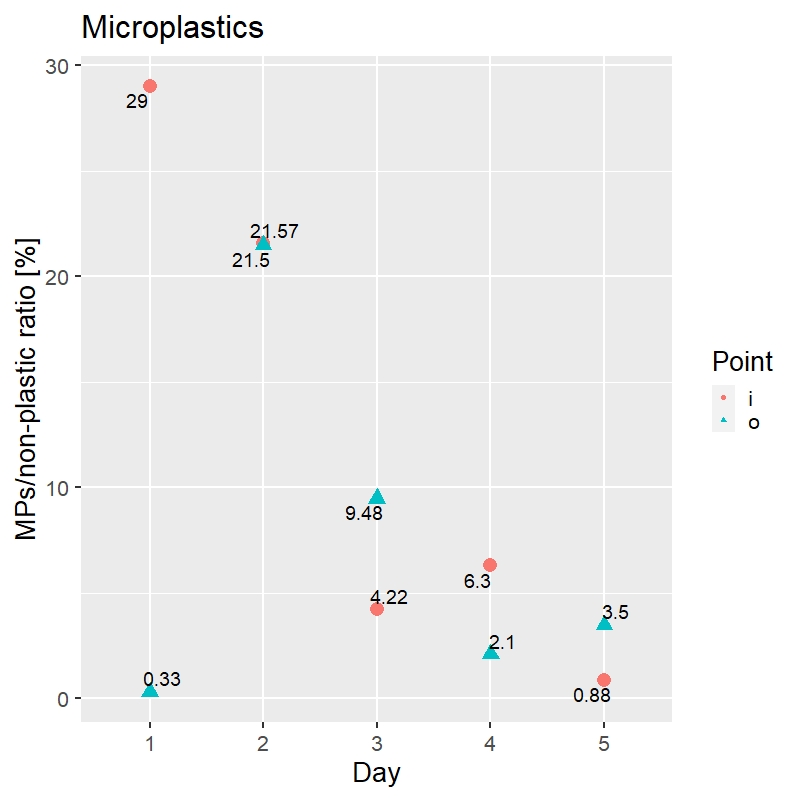


Figure S13. MP/non-plastic ratio over the investigated period at the plant’s inlet and outlet.

Table S3 and Table S4 summarize the non-blank corrected mean MP counts and mass per sample, along with the LOQ for each single identified plastic polymer. In those cases where a polymer was detected only in the drinking water samples, the LOQ was set to 0.

The mean values in Tables S3 and S4 were calculated by normalizing the MP counts or mass in the active area (deposition of 25 µL) of each field blank and drinking water sample by the total volume added upon the samples’ reconstitution (1000 µL). The means were then calculated among the replicates of each drinking water sample (n=3) and field blanks (n=10, for the LOQ).

| **Polymer** | **Raw MP counts per polymer [N/L]** | | | | | | | | | | **LOQ [N/L]** |
| --- | --- | --- | --- | --- | --- | --- | --- | --- | --- | --- | --- |
|  | **D1i** | **D1o** | **D2i** | **D2o** | **D3i** | **D3o** | **D4i** | **D4o** | **D5i** | **D5o** |  |
| ABS | 0.013 | 0 | 0 | 0 | 0 | 0 | 0 | 0 | 0 | 0 | 0 |
| Nylon 11 | 0.200 | 0.091 | 0.160 | 0.944 | 0.013 | 0.136 | 0.085 | 0.266 | 0.079 | 0.226 | 0 |
| Nylon 12 | 0.464 | 0.170 | 0.680 | 1.219 | 0.118 | 0.608 | 0.576 | 0.559 | 0.480 | 0.800 | 0.436 |
| Nylon 6 | 0.025 | 0.013 | 0.066 | 0.169 | 0.105 | 0.963 | 0.043 | 0.053 | 0.026 | 0.013 | 0.289 |
| Nylon 6,10 | 0.328 | 0.169 | 0.066 | 2.774 | 0.026 | 0.300 | 1.472 | 0.360 | 0.053 | 0.067 | 1.160 |
| Nylon 6,12 | 0.051 | 0 | 0 | 0 | 0 | 0 | 0.011 | 0 | 0 | 0 | 0 |
| Nylon 6,9 | 0.038 | 0 | 0 | 0.127 | 0 | 0.010 | 0.021 | 0 | 0.013 | 0 | 0 |
| PA resin | 3.357 | 0.053 | 0.013 | 1.293 | 0.013 | 0.032 | 0.096 | 0 | 0 | 0 | 0.311 |
| PB | 0.139 | 0 | 0.013 | 0.013 | 0 | 0 | 0 | 0.013 | 0 | 0 | 0 |
| PBA | 0 | 0 | 0 | 0.027 | 0 | 0 | 0.011 | 0 | 0 | 0 | 0 |
| PBT | 0 | 0 | 0.026 | 0 | 0 | 0 | 0 | 0 | 0 | 0 | 0 |
| PEG | 1.381 | 0 | 0 | 0.498 | 0.039 | 0 | 0.043 | 0.080 | 0 | 0 | 0.037 |
| Poly-acenaphthylene | 0 | 0 | 0 | 0 | 0 | 0 | 0 | 0 | 0.013 | 0 | 0 |
| Poly-acrylamide | 0 | 0 | 0.013 | 0.080 | 0.496 | 0.190 | 0.021 | 0.026 | 0.013 | 0.320 | 0.077 |
| Poly-acrylamide-co-acrylic acid | 0.012 | 0.026 | 0 | 0.320 | 1.093 | 0.414 | 0.128 | 0.080 | 0.013 | 0.147 | 0.078 |
| Poly-acrylic acid | 0 | 0.013 | 0.013 | 0.027 | 0.144 | 0.155 | 0 | 0.013 | 0 | 0 | 0 |
| Poly-acrylonitrile | 0 | 0 | 0 | 0 | 0 | 0.011 | 0 | 0 | 0 | 0 | 0 |
| Poly-caprolactone triol | 0 | 0.013 | 0.013 | 0.040 | 0.013 | 0 | 0 | 0.013 | 0 | 0 | 0 |
| Poly-isoprene | 0.013 | 0 | 0 | 0 | 0 | 0 | 0.032 | 0 | 0 | 0 | 0 |
| Poly-oxazoline | 0.013 | 0 | 0 | 0 | 0 | 0 | 0 | 0 | 0 | 0 | 0 |
| Poly-norbornene | 0 | 0 | 0 | 0 | 0.027 | 0.052 | 0 | 0 | 0 | 0.040 | 0 |
| Poly-vinylacetate | 0 | 0 | 0 | 0 | 0 | 0 | 0 | 0.013 | 0 | 0 | 0 |
| Poly-vinylalcohol | 0 | 0 | 0 | 0 | 0 | 0 | 0 | 0 | 0 | 0.013 | 0 |
| PMMA | 0.012 | 0 | 0 | 0 | 0 | 0 | 0 | 0 | 0.078 | 0 | 0 |
| PP | 0.038 | 0.040 | 0.040 | 0.080 | 0.013 | 0.064 | 0 | 0 | 0 | 0.040 | 0.031 |
| PP-C | 0 | 0 | 0 | 0.013 | 0 | 0 | 0 | 0 | 0 | 0 | 0 |
| PP-PB | 0 | 0.013 | 0 | 0 | 0 | 0.011 | 0 | 0 | 0 | 0 | 0 |
| PU | 0.089 | 0 | 0 | 0 | 0 | 0 | 0 | 0 | 0 | 0 | 0 |
| PVC | 0.038 | 0.080 | 0.078 | 0 | 0.078 | 0 | 0 | 0 | 0 | 0 | 0.076 |
| Techtron PPS | 0.013 | 0 | 0 | 0 | 0 | 0 | 0 | 0 | 0 | 0 | 0 |

Table S3. MP counts per L (N/L) for each identified plastic polymer. Values for the drinking water samples were not blank-corrected.

| **Polymer** | **Raw MP mass per polymer [pg/L]** | | | | | | | | | | | **LOQ [pg/L]** | |
| --- | --- | --- | --- | --- | --- | --- | --- | --- | --- | --- | --- | --- | --- |
|  | **D1i** | **D1o** | **D2i** | **D2o** | **D3i** | **D3o** | **D4i** | **D4o** | **D5i** | **D5o** |  | |  |
| ABS | <0.001 | 0.00 | 0.00 | 0.00 | 0.00 | 0.00 | 0.00 | 0.00 | 0.00 | 0.00 | 0.00 | |  |
| Nylon 11 | 0.090 | 0.016 | 0.015 | 0.129 | <0.001 | 0.020 | 0.014 | 0.055 | 0.073 | 0.527 | 0.00 | |  |
| Nylon 12 | 0.659 | 0.224 | 0.045 | 0.245 | 0.084 | 0.089 | 0.087 | 0.041 | 0.064 | 0.039 | 0.003 | |  |
| Nylon 6 | 0.007 | 0.124 | 0.750 | 0.137 | 0.094 | 2.243 | 0.018 | 1.216 | 0.034 | 0.279 | 0.450 | |  |
| Nylon 6,10 | 0.656 | 0.066 | 0.077 | 0.539 | 0.035 | 0.117 | 0.203 | 0.102 | 0.089 | 0.005 | 0.055 | |  |
| Nylon 6,12 | 0.045 | 0.00 | 0.00 | 0.00 | 0.00 | 0.00 | 0.001 | 0.00 | 0.00 | 0.00 | 0.00 | |  |
| Nylon 6,9 | 0.011 | 0.00 | 0.00 | 0.038 | 0.00 | 0.041 | <0.001 | 0.00 | 0.016 | 0.00 | 0.00 | |  |
| PA resin | 22.366 | 0.010 | <0.001 | 0.247 | 0.001 | 0.001 | 0.033 | 0.00 | 0.00 | 0.00 | 0.146 | |  |
| PB | 0.044 | 0.00 | 0.002 | <0.001 | 0.00 | 0.00 | 0.00 | <0.001 | 0.00 | 0.00 | 0.00 | |  |
| PBA | 0.00 | 0.00 | 0.00 | 0.004 | 0.00 | 0.00 | 0.003 | 0.00 | 0.00 | 0.00 | 0.00 | |  |
| PBT | 0.00 | 0.00 | 0.432 | 0.00 | 0.00 | 0.00 | 0.00 | 0.00 | 0.00 | 0.00 | 0.00 | |  |
| PE-C | 0.00 | 0.00 | 0.00 | 0.00 | 0.00 | 0.00 | 0.00 | 0.00 | 0.00 | 0.00 | 0.00 | |  |
| PEG | 3.004 | 0.00 | 0.00 | 0.098 | <0.001 | 0.00 | 0.011 | 0.022 | 0.00 | 0.00 | <0.001 | |  |
| Poly-acenaphthylene | 0.00 | 0.00 | 0.00 | 0.00 | 0.00 | 0.00 | 0.00 | 0.00 | 0.001 | 0.00 | 0.00 | |  |
| Poly-acrylamide | 0.00 | 0.00 | 0.287 | 1.413 | 0.977 | 82.338 | 1.394 | 3.498 | 0.006 | 1.036 | 0.011 | |  |
| Poly-acrylamide-co-acrylic acid | 0.003 | 0.400 | 0.00 | 0.105 | 7.038 | 53.931 | 3.079 | 0.051 | <0.001 | 0.053 | 0.111 | |  |
| Poly-acrylic acid | 0.00 | 0.014 | 2.235 | 0.012 | 0.035 | 0.027 | 0.00 | 0.007 | 0.00 | 0.00 | 0.00 | |  |
| Poly-acrylonitrile | 0.00 | 0.00 | 0.00 | 0.00 | 0.00 | 0.011 | 0.00 | 0.00 | 0.00 | 0.00 | 0.00 | |  |
| Poly-caprolactone triol | 0.00 | 0.006 | 0.001 | 0.006 | 0.003 | 0.00 | 0.00 | 0.003 | 0.00 | 0.00 | 0.00 | |  |
| Poly-1,4cyclohexanedimethylene terephthalate-co-ethylene terephthalate | 0.00 | 0.00 | 0.053 | 0.00 | 0.00 | 0.00 | 0.00 | 0.00 | 0.00 | 0.00 | 0.00 | |  |
| Poly-isoprene | 0.004 | 0.00 | 0.00 | 0.00 | 0.00 | 0.00 | 0.003 | 0.00 | 0.00 | 0.00 | 0.00 | |  |
| Poly-oxazoline | <0.001 | 0.00 | 0.00 | 0.00 | 0.00 | 0.00 | 0.00 | 0.00 | 0.00 | 0.00 | 0.00 | |  |
| Poly-norbornene | 0.00 | 0.00 | 0.00 | 0.00 | 0.002 | 0.011 | 0.00 | 0.00 | 0.00 | 0.001 | 0.00 | |  |
| Poly-vinylacetate | 0.00 | 0.00 | 0.00 | 0.00 | 0.00 | 0.00 | 0.00 | 0.034 | 0.00 | 0.00 | 0.00 | |  |
| Poly-vinylalcohol | 0.00 | 0.00 | 0.00 | 0.00 | 0.00 | 0.00 | 0.00 | 0.00 | 0.00 | <0.001 | 0.00 | |  |
| PMMA | 1.459 | 0.00 | 0.00 | 0.00 | 0.00 | 0.00 | 0.00 | 0.00 | 0.028 | 0.00 | 0.00 | |  |
| PP | 0.014 | 0.038 | 0.835 | <0.001 | 0.025 | 0.003 | 0.00 | 0.00 | 0.00 | 0.014 | 0.002 | |  |
| PP-C | 0.00 | 0.00 | 0.00 | <0.001 | 0.00 | 0.00 | 0.00 | 0.00 | 0.00 | 0.00 | 0.00 | |  |
| PP-PB | 0.00 | 0.006 | 0.00 | 0.00 | 0.00 | <0.001 | 0.00 | 0.00 | 0.00 | 0.00 | 0.00 | |  |
| PS | 0.049 | 0.025 | 6.615 | 0.001 | 0.053 | 0.029 | 0.123 | 0.012 | 5.207 | 0.123 | 0.043 | |  |
| PU | 0.006 | 0.00 | 0.00 | 0.00 | 0.00 | 0.00 | 0.00 | 0.00 | 0.00 | 0.00 | 0.00 | |  |
| PVC | 0.145 | 0.023 | 0.056 | 0.00 | 2.673 | 0.00 | 0.00 | 0.00 | 0.00 | 0.00 | 0.007 | |  |
| Techtron PPS | 0.001 | 0.00 | 0.00 | 0.00 | 0.00 | 0.00 | 0.00 | 0.00 | 0.00 | 0.00 | 0.00 | |  |

Table S4. MP mass per L (pg/L) for each identified plastic polymer. Values for the drinking water samples were not blank-corrected.

Figure S14 is a scheme of a pipe where MP breaking occurs.


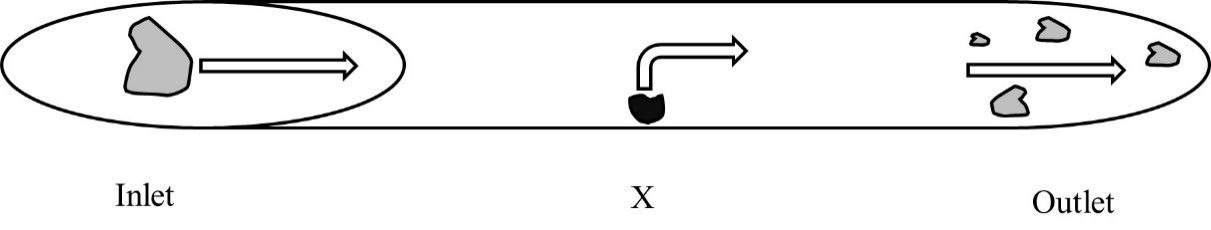
Figure S14. Scheme of a pipe where a large MP breaks down after entering the inlet. It creates four smaller MPs at the outlet, increasing the MP counts abundance at the exit, but with no increase in the MP mass abundance. However, if an MP enters the pipe from point X, the MP mass abundance also increases.

Small MPs can be produced due to mechanical stress from a larger MP entering the inlet. If the plant had a MP counts retaining efficiency of 100%, no MPs would be released into the distribution network. However, since the removal efficiency proved to be lower, some of the smaller MPs could eventually be found in the outlet samples. According to the MP counts, an increase in the MP abundance occurred in this case. On the contrary, the MP mass estimate would decrease, since the mass of the original MP at the inlet was equal to the sum of the mass of *all* the smaller MPs produced *via* breaking, including those retained by the plant. An increase in the MP mass abundance at the outlet could occur if additional MPs were released by the plant’s plastic elements located between the inlet and outlet (black MP in point X), considering the waterworks as a semi-closed system delimited by the inlet and outlet. Since the sampling took about 5 hours per day, the quantitative results for the MP abundance could be considered as mean values taking into account possible variations of the water flux in the plant.

## Polymer composition

Tables S5 and S6 report on the polymer composition according to, respectively, the MP counts and MP mass estimate at the inlet and outlet in the investigated period.

| **Inlet frequency [%]** | | | | | | | |
| --- | --- | --- | --- | --- | --- | --- | --- |
| **Day** | **Other** | **PA** | **PB** | **Poly-acrylics** | **Poly-vinyls** | **PP** | **PU** |
| 1 | 77.73 | 17.92 | 2.57 | 0.00 | 0.00 | 0.13 | 1.64 |
| 2 | 1.73 | 90.02 | 5.05 | 2.05 | 0.00 | 1.15 | 0.00 |
| 3 | 2.42 | 0.87 | 0.00 | 96.17 | 0.00 | 0.54 | 0.00 |
| 4 | 4.07 | 91.25 | 0.48 | 4.20 | 0.00 | 0.00 | 0.00 |
| 5 | 11.97 | 88.03 | 0.00 | 0.00 | 0.00 | 0.00 | 0.00 |
| **Mean** | 41.13 | 39.34 | 1.74 | 16.73 | 0.00 | 0.24 | 0.82 |
| **Outlet frequency [%]** | | | | | | | |
| **Day** | **Other** | **PA** | **PB** | **Poly-acrylics** | **Poly-vinyls** | **PP** | **PU** |
| 1 | 9.45 | 64.54 | 0.00 | 9.72 | 0.00 | 16.28 | 0.00 |
| 2 | 8.81 | 85.11 | 0.47 | 4.84 | 0.00 | 0.76 | 0.00 |
| 3 | 1.97 | 62.05 | 0.00 | 34.31 | 0.00 | 1.66 | 0.00 |
| 4 | 1.23 | 90.68 | 1.23 | 5.59 | 1.26 | 0.00 | 0.00 |
| 5 | 2.41 | 57.06 | 0.00 | 39.21 | 0.78 | 0.54 | 0.00 |
| **Mean** | 6.27 | 77.88 | 0.38 | 14.25 | 0.19 | 1.02 | 0.00 |

Table S5. MP polymer frequency over the five investigated days at the plant’s inlet and outlet (counts).

| **Inlet frequency [%]** | | | | | | | |
| --- | --- | --- | --- | --- | --- | --- | --- |
| **Day** | **Other** | **PA** | **PB** | **Poly-acrylics** | **Poly-vinyls** | **PS** | **PU** |
| 1 | 22.84 | 76.74 | 0.09 | 0.00 | 0.00 | 0.31 | 0.01 |
| 2 | 0.37 | 8.28 | 2.94 | 21.04 | 0.00 | 67.37 | 0.00 |
| 3 | 0.06 | 2.27 | 0.00 | 97.11 | 0.00 | 0.55 | 0.00 |
| 4 | 0.09 | 3.13 | 0.01 | 95.90 | 0.00 | 0.87 | 0.00 |
| 5 | 0.50 | 6.57 | 0.00 | 0.30 | 0.00 | 92.63 | 0.00 |
| **Mean** | 10.44 | 37.40 | 0.56 | 19.36 | 0.00 | 17.61 | < 0.01 |
| **Outlet frequency [%]** | | | | | | | |
| **Day** | **Other** | **PA** | **PB** | **Poly-acrylics** | **Poly-vinyls** | **PS** | **PU** |
| 1 | 0.30 | 39.10 | 0.00 | 58.30 | 0.00 | 2.30 | 0.00 |
| 2 | 3.44 | 42.39 | 0.10 | 54.07 | 0.00 | 0.00 | 0.00 |
| 3 | 0.06 | 37.77 | 0.00 | 61.88 | 0.00 | 0.28 | 0.00 |
| 4 | 0.48 | 27.11 | < 0.01 | 71.95 | 0.24 | 0.22 | 0.00 |
| 5 | 0.03 | 28.84 | 0.00 | 67.37 | 0.01 | 3.75 | 0.00 |
| **Mean** | 0.56 | 33.81 | 0.01 | 64.82 | 0.08 | 0.72 | 0.00 |

Table S6. MP polymer frequency over the five investigated days at the plant’s inlet and outlet (mass estimate).

# Morphological analysis

Table S7 shows the frequency of the plastic particles according to the five chosen length ranges, as calculated by the Raman microscope software.

| **Inlet frequency [%]** | | | | | |
| --- | --- | --- | --- | --- | --- |
| **Day** | **1 – 5 µm** | **5 – 10 µm** | **10 – 20 µm** | **20 – 50 µm** | **50+ µm** |
| 1 | 82.20 | 11.30 | 5.37 | 0.72 | 0.36 |
| 2 | 83.70 | 9.39 | 5.71 | 1.22 | 0.00 |
| 3 | 80.90 | 12.60 | 4.11 | 2.41 | 0.00 |
| 4 | 83.40 | 12.70 | 2.70 | 0.76 | 0.41 |
| 5 | 87.40 | 10.30 | 2.06 | 0.00 | 0.26 |
| **Mean** | 83.29 | 11.89 | 3.62 | 0.89 | 0.31 |
| **Outlet frequency [%]** | | | | | |
| **Day** | **1 – 5 µm** | **5 – 10 µm** | **10 – 20 µm** | **20 – 50 µm** | **50+ µm** |
| 1 | 89.80 | 6.14 | 3.07 | 1.02 | 0.00 |
| 2 | 93.50 | 5.57 | 0.50 | 0.30 | 0.10 |
| 3 | 91.80 | 6.60 | 0.94 | 0.67 | 0.00 |
| 4 | 93.50 | 4.90 | 1.09 | 0.54 | 0.00 |
| 5 | 93.60 | 4.87 | 0.78 | 0.78 | 0.00 |
| **Mean** | 92.70 | 5.68 | 0.99 | 0.58 | 0.03 |

Table S7. MP length ranges frequency over Days 1 – 5 at the plant’s inlet and outlet with mean values. Values not corrected for blanks contamination.

Figure S15 is a boxplot showing the length distribution of the analysed MPs in the samples.


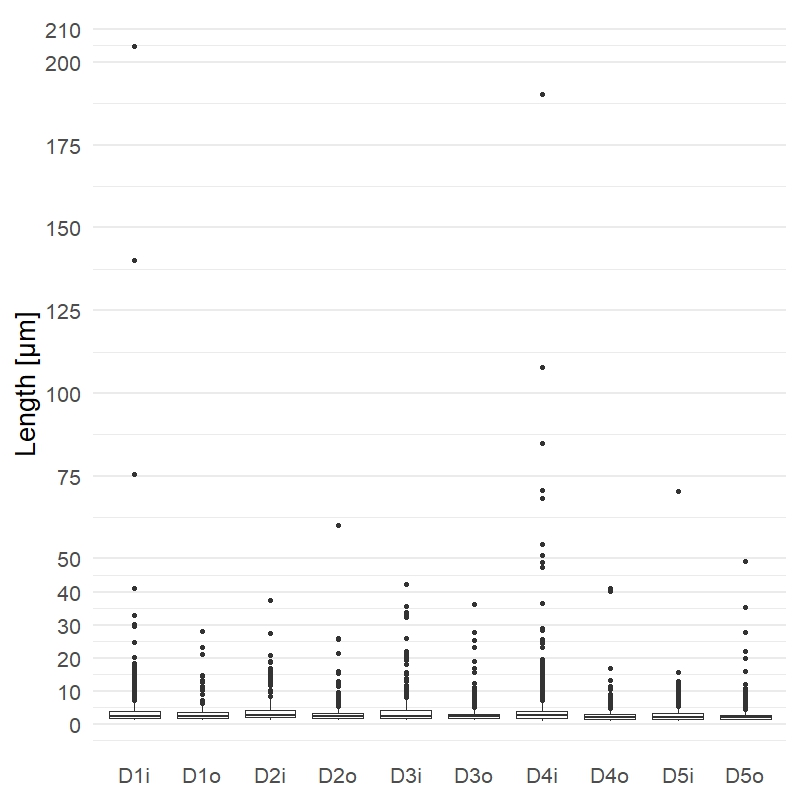


Figure S15. Boxplot showing the length distribution of all identified MPs (values not corrected for blank contamination). The median is represented by the thick black line in the boxes, while the points indicate the outliers.

Figure S16 illustrates the counts and percentage of MP fibres and fragments in the samples. MPs with a length greater or equal to 3 times the width were considered fibres.


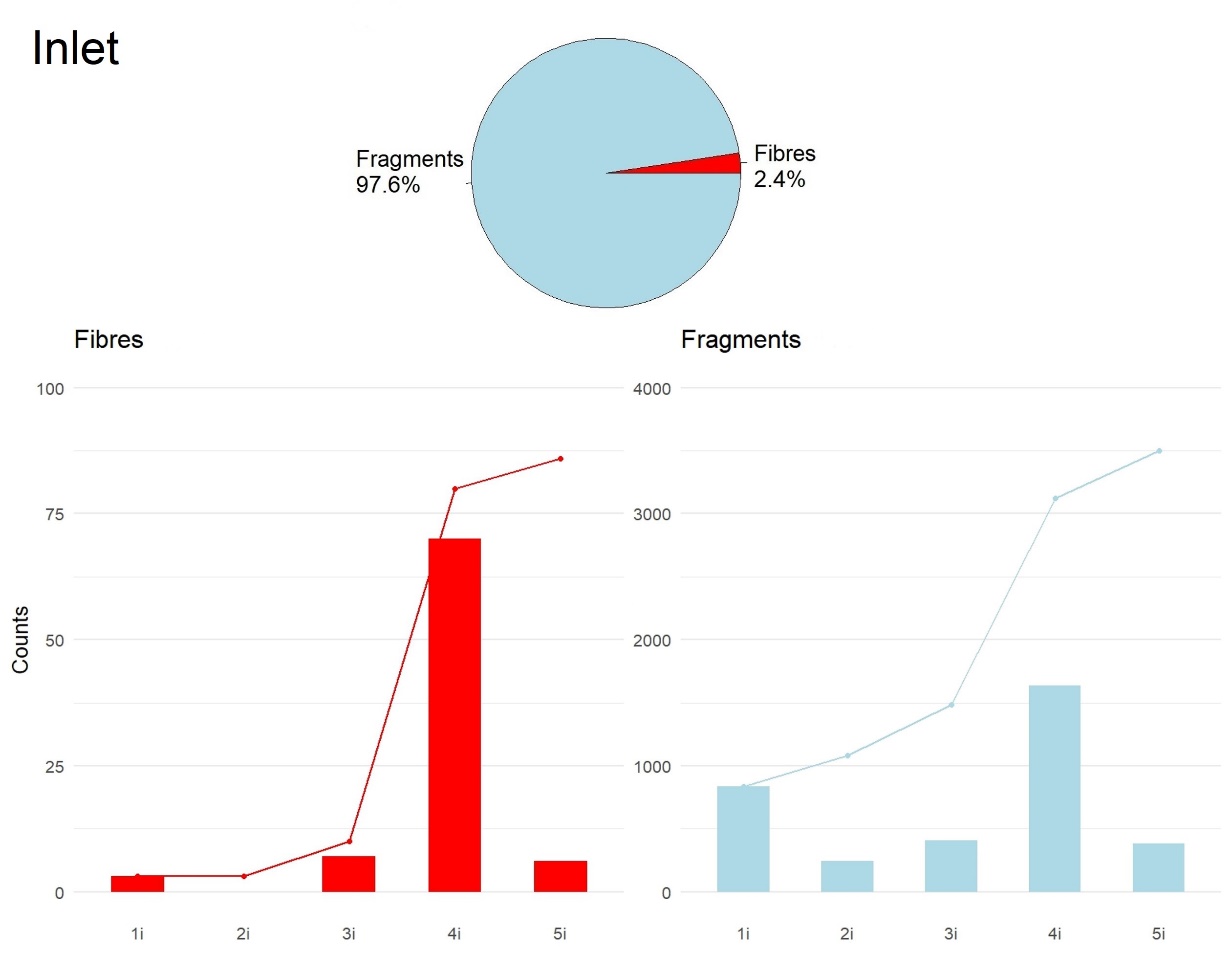


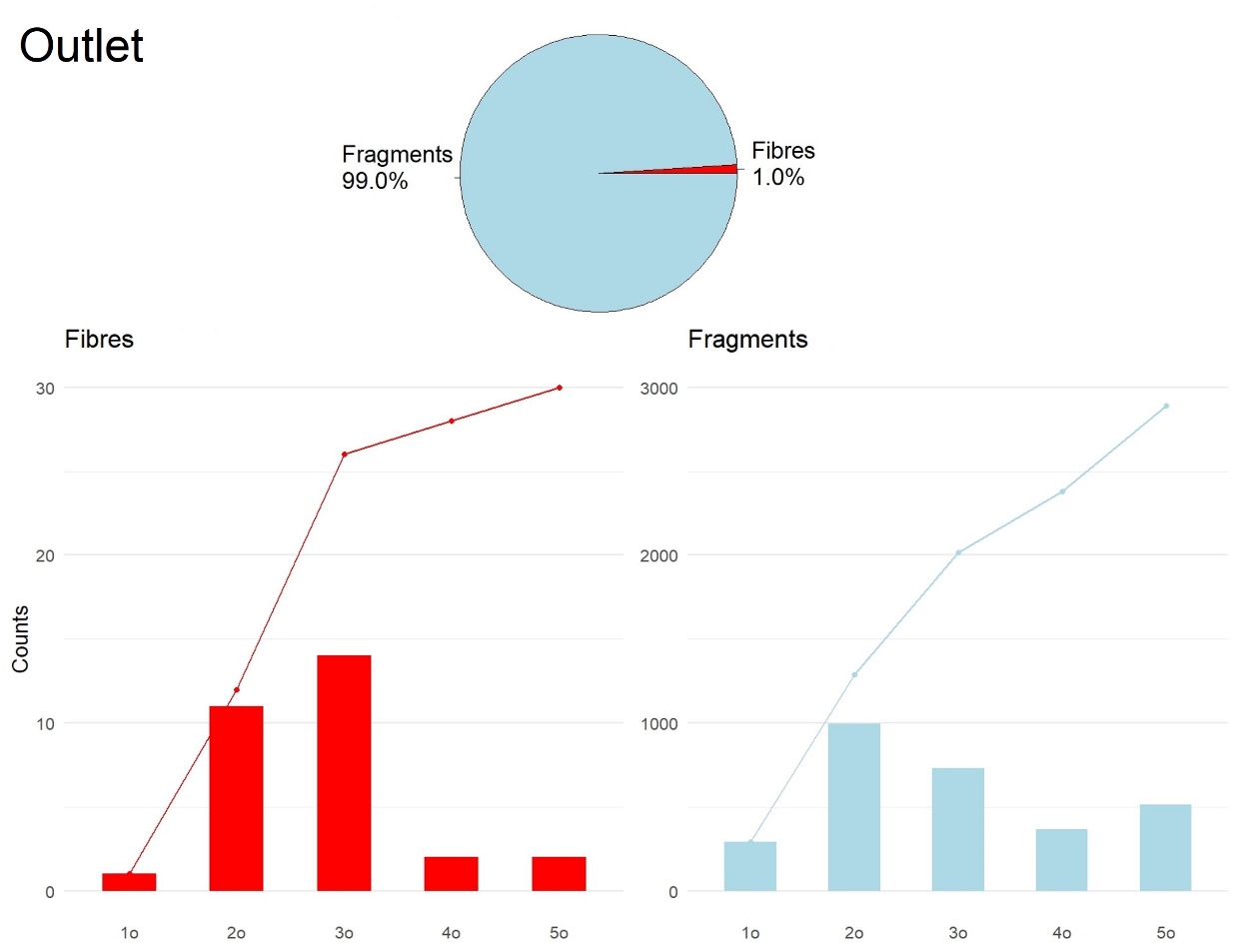


Figure S16. Total counts of fibres and fragments at the inlet and outlet over the investigated period. The lines indicate the cumulated counts. Values not corrected for blank contamination.

# Raman spectra from environmental plastic particles

Below are examples of experimental Raman spectra acquired during the sample analysis with the related reference spectra. For clarity, the spectral range was cut at 1100 cm^-1^ to exclude the signals from the Si substrate. In the caption of each figure, the HQ between 0 and 1 of the spectral recognition is also reported.

## Particles above 1 µm (MPs)

*Figure S17. Raman spectrum of PBA (category PB, HQ 0.89).*

Figure S18. Raman spectrum of Poly(ethylene-co-ethyl acrylate), for simplicity called PE in the discussion (HQ 0.97).


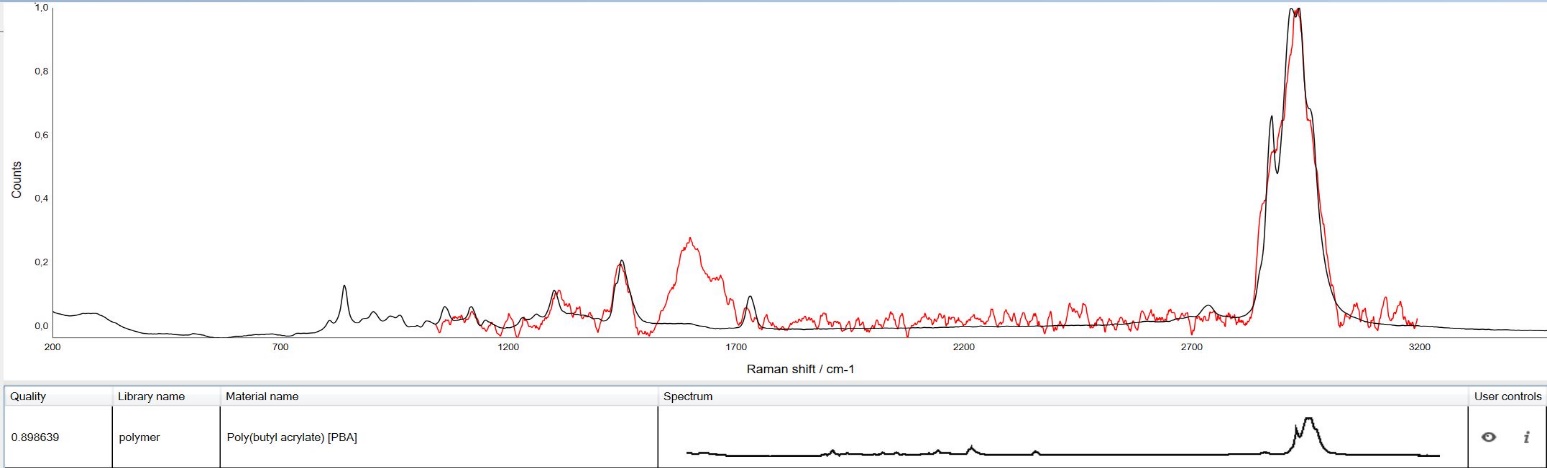


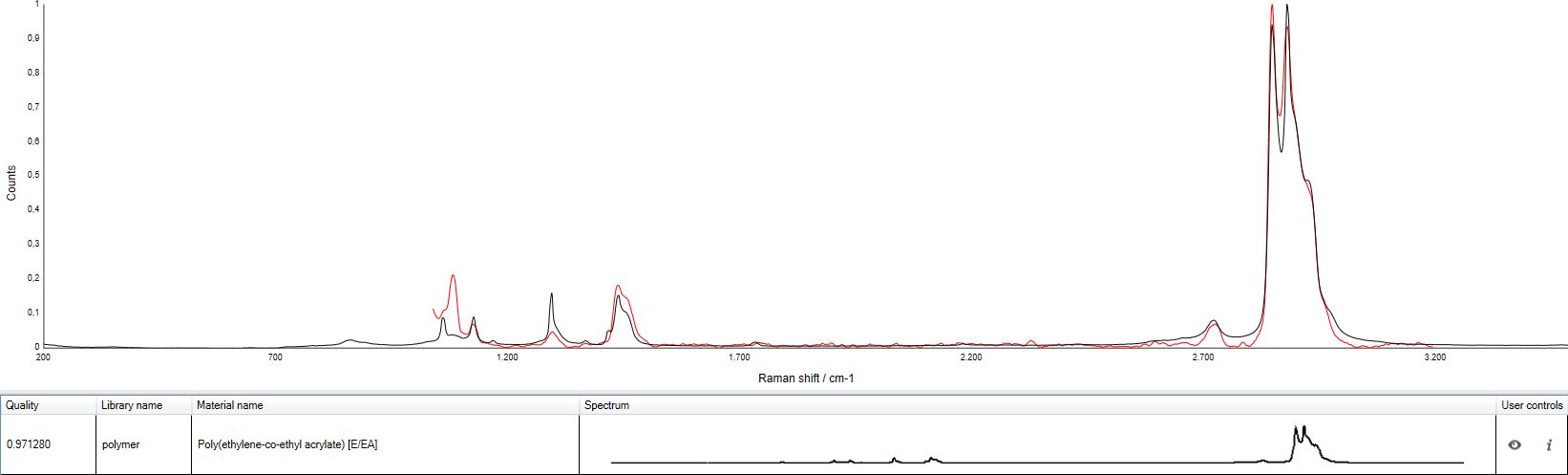


*
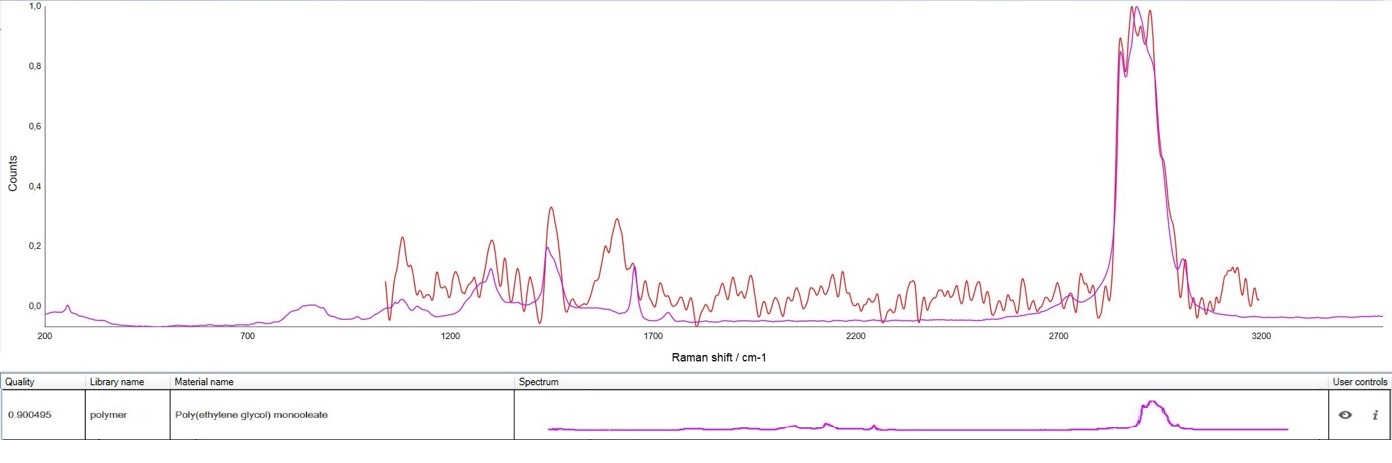
Figure S19. Raman spectrum of PEG (category Other, HQ 0.90).*

Figure S20. Raman spectrum of Poly(acrylamide-co-acrylic acid) (category poly-acrylics, HQ 0.82).


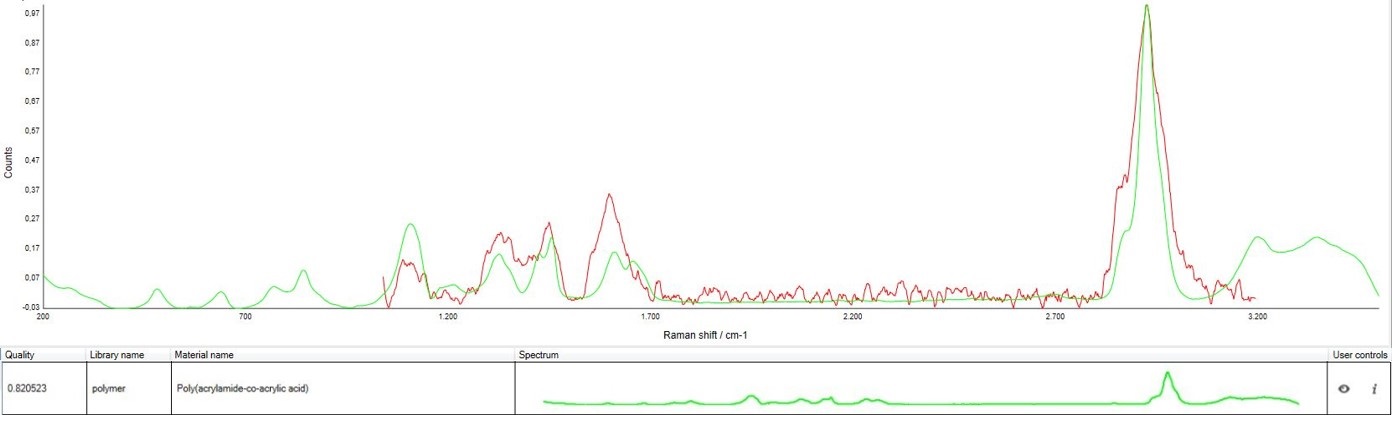

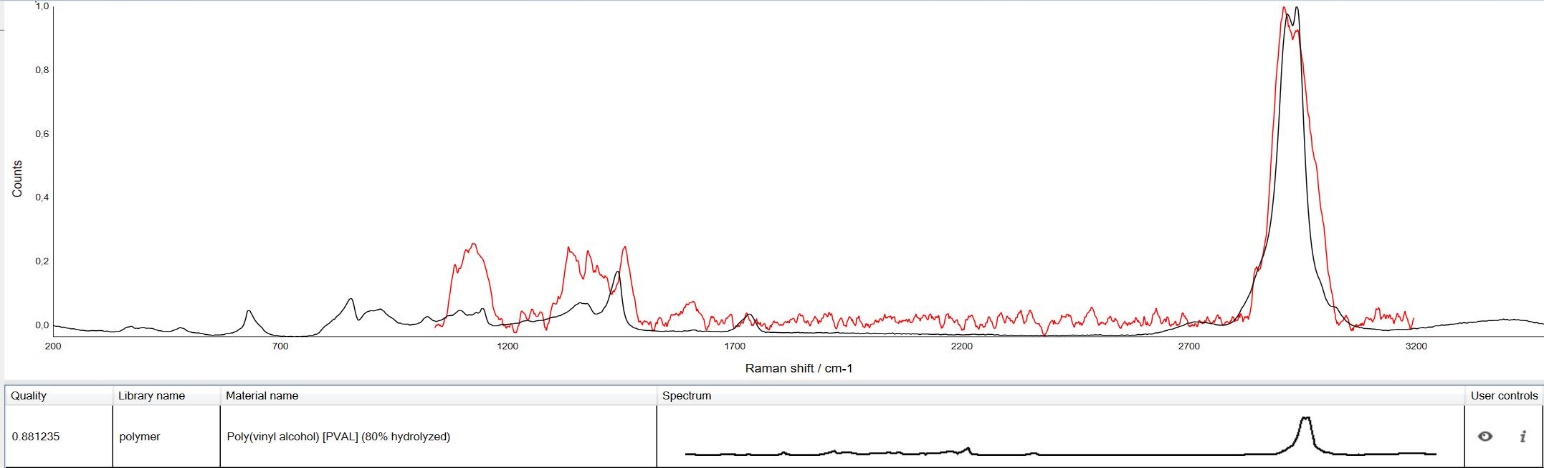


Figure S21. Raman spectrum of Poly(vinyl alcohol) (category poly-vinyls, HQ 0.88).


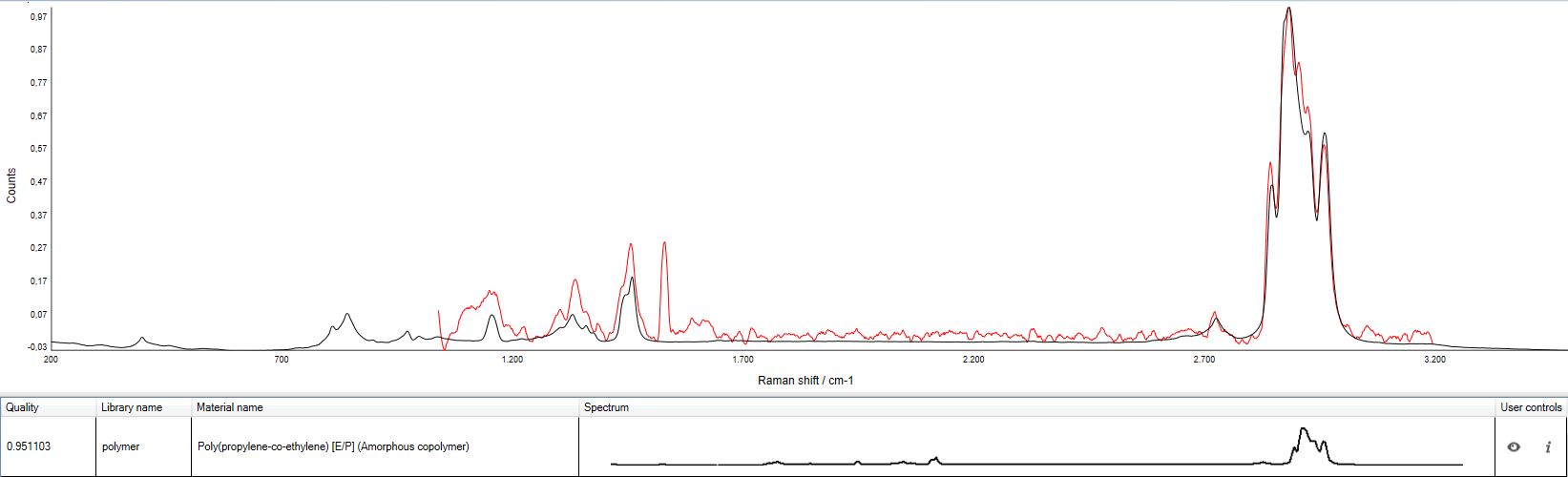


Figure S22. Raman spectrum of PP (HQ 0.95).

Figure S23
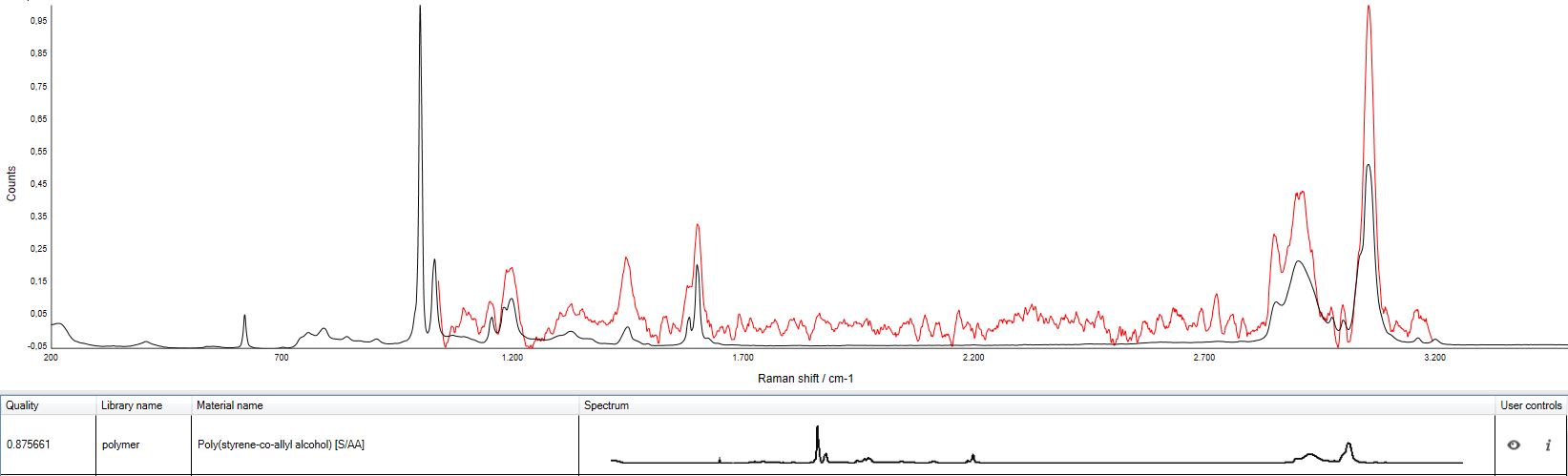
. Raman spectrum of PS (HQ 0.87).


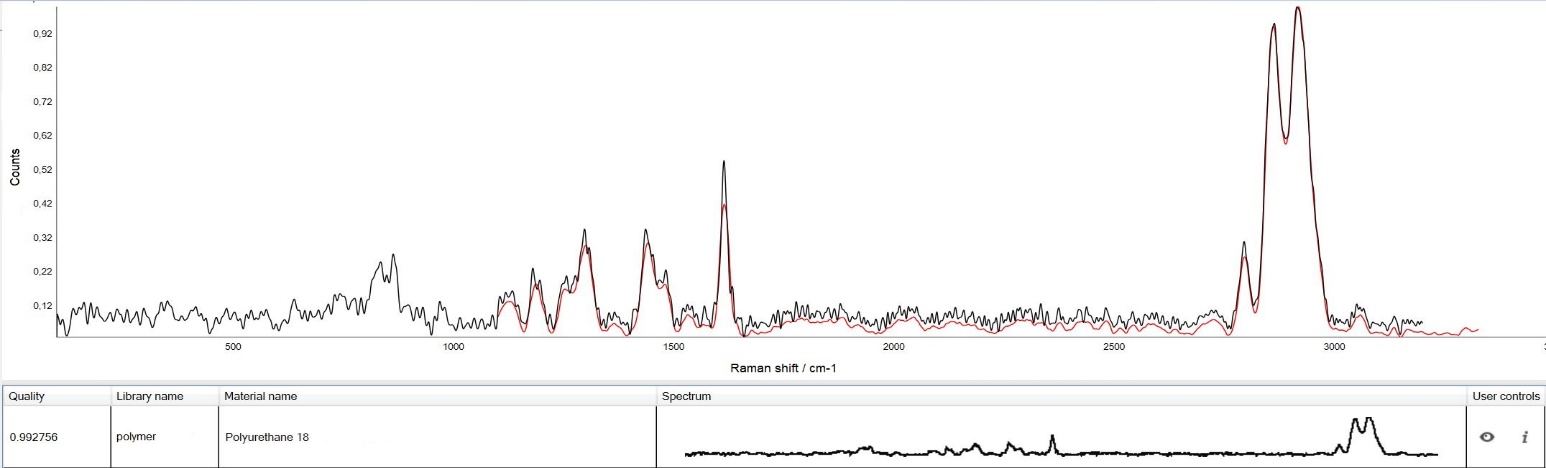


Figure S24. Raman spectrum of PU (HQ 0.99).

## Particles below 1 µm (NPs)
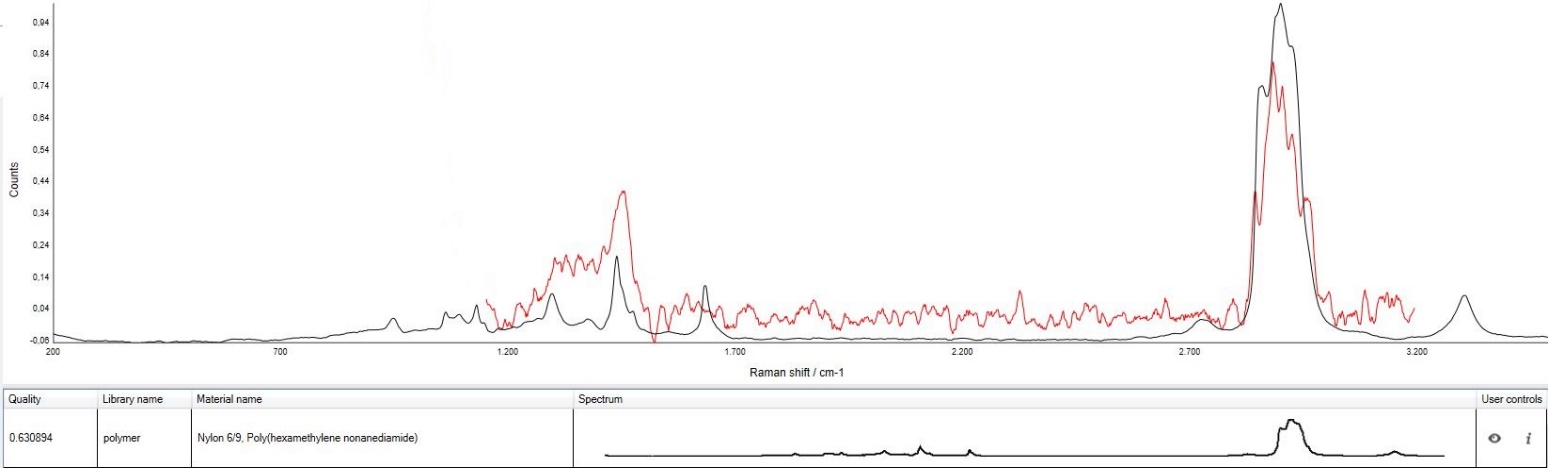


Figure S25. Raman spectrum of Nylon 6,9 nanoparticle (HQ 0.63).

*
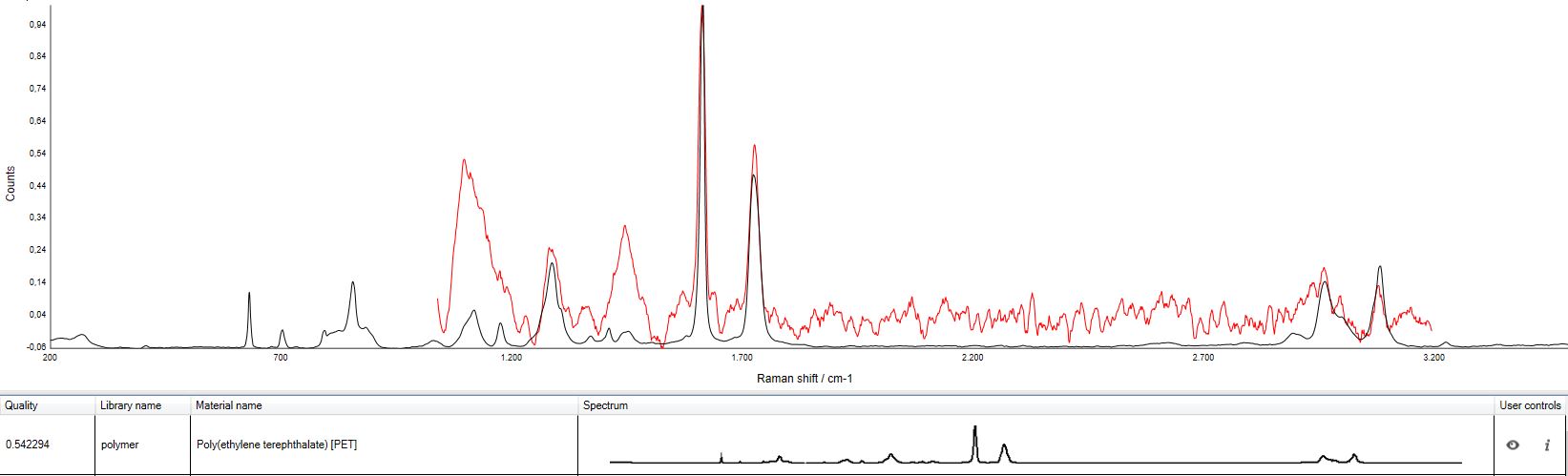
Figure S26. Raman spectrum of PET nanoparticle (HQ 0.54).*


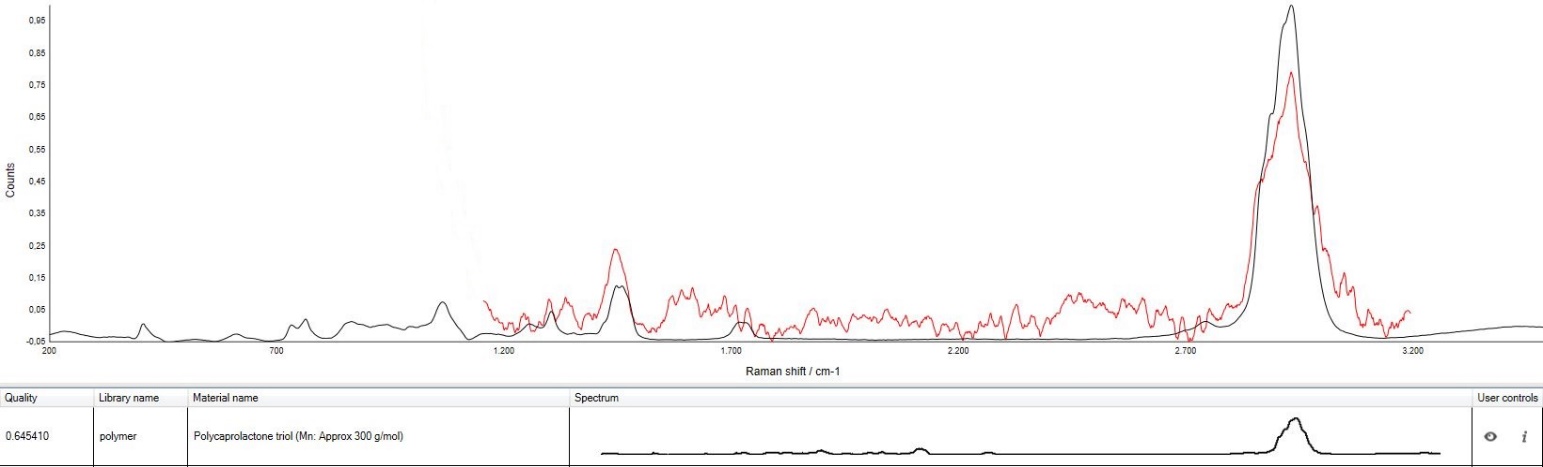


Figure S27. Raman spectrum of Polycaprolactone triol nanoparticle (HQ 0.64).

# References

[1] von der Esch E., Kohles A. J., Anger P. M., Hoppe R., Niessner R., Elsner M. Ivleva N. P., TUM-ParticleTyper: A detection and quantification tool for automated analysis of (Microplastic) particles and fibers. PLOS ONE 15, 6, e0234766 (2020), <https://doi.org/10.1371/journal.pone.0234766>

[2] Schymanski D., Oßmann B. E., Benismail N., Boukerma K, Dallmann G., von der Esch E., Fischer D., Fischer F., Gilliland D., Glas K., Hofmann T., Käppler A., Lacorte S., Marco J., Rakwe E. L. M., Weisser J., Witzig C., Zumbülte N., Ivleva N. P., Analysis of microplastics in drinking water and other clean water samples with micro-Raman and micro-infrared spectroscopy: minimum requirements and best practice guidelines., Anal. and Bioanal. Chem. 413, 5969 –5994 (2021), <https://doi.org/10.1007/s00216-021-03498-y>
